# Functional Connectivity Patterns Following Mild Traumatic Brain Injury and the Association With Longitudinal Cognitive Function

**DOI:** 10.1002/hbm.70237

**Published:** 2025-05-27

**Authors:** Goretti España‐Irla, Emma M. Tinney, Meishan Ai, Mark Nwakamma, Timothy P. Morris

**Affiliations:** ^1^ Department of Physical Therapy, Movement, & Rehabilitation Sciences Northeastern University Boston Massachusetts USA; ^2^ Center for Cognitive & Brain Health, Northeastern University Boston Massachusetts USA; ^3^ Department of Psychology Northeastern University Boston Massachusetts USA; ^4^ Department of Applied Psychology Northeastern University Boston Massachusetts USA

## Abstract

Functional magnetic resonance imaging (fMRI) has revealed subtle neuroplastic changes in brain networks following mild traumatic brain injury (mTBI), even when standard clinical imaging fails to detect abnormalities. However, prior findings have been inconsistent, in part due to methodological differences and high researcher degrees of freedom in region‐based analyses, which often rely on predefined hypotheses and overlook complex, distributed connectivity patterns. Here, we apply an unbiased, data‐driven multi‐voxel pattern analysis (MVPA) to examine whole‐brain functional connectivity differences in a large cohort of individuals with acute mTBI. Unlike conventional statistical approaches, MVPA enables a data‐driven analysis of brain‐wide connectivity patterns without requiring prior assumptions about the location or nature of abnormalities, allowing for the identification of the most informative features. This approach provides an exploratory characterization of whole‐brain functional connectivity patterns and their relationship with cognitive recovery, offering new insights into the neural mechanisms underlying post‐injury outcomes. A total of 265 adults (87 women) between 18 and 83 years old with Glasgow Coma Scale (GCS) scores of 13–15 were included in this analysis. Two replicate samples (*n* = 165, *n* = 155), with similar demographic characteristics, were also included. Data were collected as part of the prospective multi‐center Transforming Research and Clinical Knowledge in TBI (TRACK‐TBI). The goal of this study was to assess whole‐brain functional connectivity patterns using fc‐MVPA and post hoc seed‐to‐voxel analyses in a large, well‐characterized sample to determine if changes in functional connectivity can differentiate subacute mTBI (within 2 weeks of injury) from a matched group of orthopedic control subjects (*n* = 49). Additionally, we aimed to investigate whether these connectivity patterns were linked to cognitive performance at 2 weeks, 6 months, and 12 months post‐injury to better understand cognitive trajectories and recovery over time in individuals with mTBI. Voxel‐to‐voxel functional connectivity across the entire connectome revealed significant differences between TBI and no TBI in the functional connectivity patterns of 8 clusters (*p*‐voxel < 0.001, FEW cluster‐level *p* < 0.05) (*k* > 40, Fmax = 15.36), including right occipital cortex, anterior cingulate gyrus, inferior and middle temporal gyrus, right thalamus, left cerebellum, and the bilateral frontal pole. These clusters belong mainly to the visual network (VIS), frontoparietal network (FPN), default mode network (DMN) and limbic network (LIM). Post hoc characterization of each significant cluster revealed by MVPA using seed‐to‐voxel analysis showed a mixed pattern of connectivity between relevant networks and subcortico‐cortical connections. After connectivity characterization, visual‐motor skills assessed with Trail Making Test (TMT) A were significantly associated with the increased anticorrelation between the inferior temporal cortex and the bilateral occipital pole (FPN‐VIS connectivity), along with decreased anticorrelations between the cerebellum and extensive areas of the somatomotor network (SMN) over 12 months post injury. Additionally, hypoconnectivity between the frontal pole (LIM) and anterior cingulate gyrus (salience network [SAL]) was associated with better executive functions performance measured by TMT‐B over 12 months post‐mTBI. In our study examining neuroplastic changes following TBI across the entire voxel‐to‐voxel functional connectome, we identified significant differences in the functional connectivity patterns of several regions known to be particularly vulnerable to injury mechanisms. Our findings highlight the complex and compensatory nature of brain network alterations after mTBI, suggesting both detrimental and adaptive changes in connectivity that affect cognitive functions. Consequently, our study provides novel evidence that specific brain networks and regions are particularly susceptible to functional connectivity changes during the acute stages of mTBI, which are related to cognitive recovery post‐injury.

## Graphical abstract

High‐resolution fMRI using multi‐voxel pattern analysis (MVPA) reveals distinct alterations in functional connectivity across multiple brain networks in acute mTBI patients. These connectivity changes are associated with cognitive performance over 12 months, highlighting both detrimental and adaptive neuroplastic changes in response to injury.

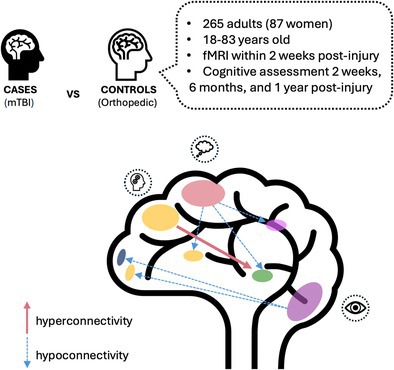

## 1 Introduction

Population‐based studies show that 50–60 million people worldwide are affected by a new traumatic brain injury (TBI) each year (Air et al. 2017). Around 60%–95% of all TBI cases are categorized as mild head injuries (Lefevre‐Dognin et al. 2021). Mild traumatic brain injuries (mTBI), also referred to as concussion, usually result from acceleration and deceleration energy forces occurring secondary to a direct blow to the head or external physical forces (Georges and M Das 2024). Most cases of mTBI are followed by clinical recovery, both physically and cognitively, shortly after the injury. Recovery typically includes the resolution of acute symptoms such as headaches, nausea, vomiting, dizziness, balance issues, fatigue, sleep disturbances, drowsiness, sensitivity to light or noise, blurred vision, memory difficulties, and trouble concentrating (McCrory et al. 2009; Nelson et al. 2018). The majority of individuals return to their normal daily routines, including work and social activities, without lasting impairments within days to weeks. However, a subset of individuals experience prolonged post‐concussive symptoms, including cognitive, emotional, somatic, and motor deficits, as well as sleep disturbances, which can persist for up to 7 years post‐injury (Brett et al. 2023; Kleiner et al. 2018; McCrea et al. 2013; Tinney et al. 2024).

Cognitive impairment is one of the most frequently reported symptoms of mTBI and has been seen to persist up to one year post‐injury (Schneider et al. 2022). Additionally, large epidemiological studies have identified mTBI as a potential risk factor for dementia (Gardner and Yaffe 2015). Concretely, mTBI can lead to impairments across various cognitive domains, including attention, memory, and/or executive functions, and may cause irritability, anxiety, or depression (Pavlovic et al. 2019). Given the vulnerability of the frontal lobes and anterior brain networks to TBI, it is not surprising that patients often exhibit significant deficits in executive functions, which are critical for higher level cognitive processes, behavioral regulation, task management, prioritizing activities, impulse control, and adaptation to change (Diamond 2013; Rabinowitz and Levin 2014).

Neuroimaging technologies such as computed tomography (CT) and conventional magnetic resonance imaging (MRI) can provide additional diagnostic and prognostic information in the clinical management of mTBI (Hu et al. 2022). However, only a small proportion of mTBI patients meet the criteria for neuroimaging. The limited use of imaging may overlook subclinical brain changes that could provide valuable prognostic information in mTBI survivors (Palacios et al. 2017). Functional magnetic resonance imaging (fMRI) holds potential for mTBI diagnosis and prognosis (Kou et al. 2010). By measuring changes in blood flow, it can reveal precise neural activity patterns in specific brain regions (Abdul Rahman et al. 2022; D'Souza et al. 2020; Johnson et al. 2012; Mayer et al. 2011; Morelli et al. 2021). Resting state functional MRI (rs‐fMRI) has revealed neuroplastic changes in brain networks following a mTBI that are subtle and subclinical, undetectable with CT imaging (Abdul Rahman et al. 2022; Beauchamp et al. 2011; D'Souza et al. 2020; Hu et al. 2022; Johnson et al. 2012; Manolakaki et al. 2009; Mayer et al. 2011; Morelli et al. 2021; Palacios et al. 2017). In healthy brains, functional connectivity within and between large‐scale brain networks is essential for adaptation, learning, and maintaining cognitive and sensorimotor functions (Mohr et al. 2016; Pascual‐Leone et al. 2005; Xu et al. 2021). Reduced within‐network connectivity is generally considered detrimental, as it may reflect the degradation of white matter integrity and impair the efficient communication necessary for specialized network functions (Varangis et al. 2019). However, following neural disruption, compensatory mechanisms may lead to increased connectivity both within and between networks where hyperconnectivity has been proposed as an adaptive response (Hillary et al. 2014, 2015; Hillary and Grafman 2017). Conversely, between‐network connectivity facilitates the integration of information across functionally specialized regions, supporting complex cognitive processes. While such integration is essential, excessive cross‐network connectivity may reduce network segregation, leading to a less efficient, undifferentiated functional organization (Varangis et al. 2019). Maintaining an optimal balance between network integration and segregation is therefore critical for preserving cognitive and behavioral functions (Cohen and D'Esposito 2016; Varangis et al. 2019; R. Wang et al. 2021). Following mTBI, functional network dynamics are disrupted due to structural alterations in both white and gray matter (Dean et al. 2015a; Jia et al. 2021; Song et al. 2021). White matter damage, often resulting from diffuse axonal injury, impairs the integrity of major fiber tracts, leading to disruptions in long‐range network communication and reduced within‐network connectivity (Jia et al. 2021). In cases of widespread white matter damage, one might observe long‐range disconnection across the brain, potentially manifesting as anticorrelations within a network, which would be detrimental to overall network function (Pandit et al. 2013). In contrast, gray matter injury, particularly focal damage, impacts localized regions, diminishing connectivity with other areas that would typically be functionally coupled, leading to a reduction in functional coherence between that region and the damaged area. The heterogeneous nature of mTBI, arising from differences in clinical presentation, time since injury, and severity, biomechanics, and anatomical location (Mayer et al. 2017), naturally gives rise to considerable variability in connectivity changes and compensatory strategies following injury (Abbas et al. 2015; Churchill et al. 2017b, 2017a; Hammeke et al. 2013; Johnson et al. 2012; Zhang et al. 2012; Zhu et al. 2015). However, the inherent variability in functional connectivity profiles is exacerbated by the multitude of methodological choices that can be made in modeling resting state functional MRI data (Morelli et al. 2021). The wide variety of methodologies has resulted in a mixed literature on functional connectivity changes after TBI. While adaptive hyperconnectivity is well‐documented in moderate and severe TBI (Hillary et al. 2014, 2015; Hillary and Grafman 2017), there is a substantive lack of consensus regarding network connectivity responses to mTBI. In a comprehensive literature review, Morelli et al. (2021) examined functional connectivity changes post‐mTBI, revealing a mix of hypoconnectivity and hyperconnectivity due to diverse mTBI mechanisms, age ranges, and time from injury to MRI scans. Several studies utilizing independent component analyses (ICA) and regions of interest (ROI) analyses have reported a consistent pattern of decreased connectivity in the anterior regions of functional networks, accompanied by increased connectivity in the posterior regions in mTBI population. This pattern has been observed in specific networks such as the default mode network (DMN) and the frontoparietal network (FPN) (D'Souza et al. 2020; Iraji et al. 2015; Mayer et al. 2015; Palacios et al. 2017; Vakhtin et al. 2013; van der Horn et al. 2017). Additionally, this pattern was consistent across other functional networks, such as the somato‐motor network (SMN), cerebellar, and limbic networks, where mixed reports of both hyperconnectivity and hypoconnectivity across studies are found (Morelli et al. 2021). To date, relatively few studies have employed functional connectivity multivariate pattern analysis (fc‐MVPA) to examine the relationship between neural functioning and individual differences in mTBI (Guell et al. 2020; Thompson et al. 2016), despite its successful application in other medical conditions, including mental health disorders (Fitzgerald et al. 2020; Visser et al. 2016) and autism spectrum disorder (Arnold Anteraper et al. 2019). A recent study applied whole‐brain fc‐MVPA to investigate functional connectivity differences in retired professional athletes with a history of multiple sport‐related concussions compared to matched controls. The analysis identified a cluster of abnormal functional connectivity in cerebellar lobule V, a region associated with the ventral attention network. Subsequent seed‐to‐voxel analyses revealed widespread cortical hyper‐ and hypo‐connectivity in the retired athletes, suggesting potential cerebellar dysfunction (Guell et al. 2020). Overall, these findings demonstrate the potential of fc‐MVPA to detect subtle, spatially distributed connectivity alterations in mTBI. By reducing methodological choices, this approach allows for a data‐driven exploration of network disruptions without requiring a priori assumptions.

Functional connectivity multivariate pattern analysis is an unbiased, data‐driven approach that can potentially address inconsistencies in mTBI imaging studies. Compared to traditional methods like ROI‐to‐ROI or seed‐to‐voxel analysis, fc‐MVPA offers a more comprehensive analysis by taking each voxel in the brain and computing the functional connectivity between that voxel and every other voxel in the brain for each subject individually (Nieto‐Castanon 2022). Subsequently, connectivity maps for each seed voxel are concatenated and further reduced dimensionality is achieved using singular value decomposition. The resulting eigenpattern scores are then used in a group‐level general linear model to test whether the difference between groups differs significantly from zero at each voxel. This process is repeated across all voxels in the brain mask, resulting in a statistical parametric map that reflects the results across the entire brain. This method significantly reduces the constraints of traditional statistical approaches, enabling exploratory analysis to identify the most crucial data features without needing predefined hypotheses about the location or nature of functional connectivity abnormalities (Guell et al. 2020; Thompson et al. 2016). Thus, through its detailed analysis of connectivity patterns, fc‐MVPA holds potential as a promising tool for identifying key biomarkers and enhancing clinical outcomes in mTBI research.

Despite the advancements and findings from various neuroimaging studies, the need for a more comprehensive and unbiased approach to understand functional connectivity changes and their correlation with long‐term outcomes in mTBI remains critical. Therefore, the purpose of this study was to evaluate whole‐brain functional connectivity patterns using fc‐MVPA in a large and well‐characterized sample to investigate where there are differences in functional connectivity between those with a sub‐acute (within 2 weeks of injury) mTBI and orthopedic controls. Second, we aimed to test if these patterns were associated with cognition at 2 weeks, 6 months, and 12 months post‐injury to better characterize cognitive trajectories and recovery in mTBI over time.

## 2 Methods

### 2.1 Standard Protocol Approvals, Registrations, and Patient Consents

These data were obtained from the open‐access Federal Interagency Traumatic Brain Injury Research informatics system (FITBIR) under a data use agreement with Northeastern University. Participants were recruited from 11 academic level 1 trauma centers across the United States within 24 h of injury, following evaluation in the emergency department or inpatient unit for TBI. Written informed consent was obtained, and the study protocol received approval from the University of California, San Francisco, and all participating site Institutional Review Boards. Outcome assessments were conducted in person at 2 weeks, 6 months, and 12 months post‐injury, with an additional phone assessment at 3 months. Data collection adhered to the TBI Common Data Elements (Maas et al. 2010), and assessments were administered and scored by trained study staff. The Glasgow Coma Scale (GCS) was used to classify injury severity upon arrival. The primary inclusion criteria for the Transforming Research and Clinical Knowledge in Traumatic Brain Injury (TRACK‐TBI) study were patients who presented to the participating centers within 24 h of injury with clinical indications for a CT scan as per the American College of Emergency Medicine/Center for Disease Control and Prevention Criteria (Jagoda et al. 2008). Exclusion criteria included incarceration, pregnancy, nonsurvivable physical trauma, psychiatric hold, debilitating mental health disorders, neurological disease, and non‐English or non‐Spanish speakers, depending on the site. Comprehensive inclusion and exclusion criteria for TRACK‐TBI were detailed in previous publications (Nelson et al. 2019; Yue et al. 2013).

### 2.2 Participants

A total of 1132 patients with mTBI met the inclusion criteria of admission GCS 13–15. Of these, 615 underwent MRI within 2 weeks (range: 10–18 days). In‐person outcome assessments were completed at 2 weeks (including an MRI, and cognitive assessment), 6 months (MRI, cognitive assessment), and 12 months post‐injury (cognitive assessment). Participant characteristics for TRACK‐TBI Siemens sub‐sample are presented in Table 1.

**TABLE 1:** TRACK‐TBI participant characteristics (Siemens sub‐sample).

	Groups	
	TBI (mean ± SD)	No TBI (mean ± SD)
Age	40.36 ± 17.00	40.60 ± 16.97
Sex	147 M, 69 F	31 M, 18 F
Education	14.00 ± 2.8	14.4 ± 2.8
Household Income	$25,000 to $34,999	$25,000 to $34,999
American Indian (%)	1%	0%
Asian (%)	2%	4%
Black/African American (%)	13%	10%
White (%)	83%	83%
Native Hawaiian or Other Pacific Islander	0%	0%
More than one/unknown (%)	1%	0%

Abbreviations: df, degrees of freedom; SD, standard deviation; TBI, traumatic brain injury.

### 2.3 fMRI Acquisition

MRI scans of 615 participants from the TRACK‐TBI study were acquired across 11 centers using 13 different 3T MRI scanners from three different manufacturers. The scan was performed within 15 days (range, 5–18) and 6 months post‐injury. A 7 min rsfMRI single shot gradient‐echo echo planar imaging (EPI) sequence was acquired (repetition time [TR] = 2000 ms, echo time [TE] = 28 ms; flip angle = 90 grad; field of view [FOV] = 220 mm; voxel size = 3.4 × 3.4 × 4.0 mm). The subjects were asked to close their eyes, relax, not focus their attention on anything specific, and not fall asleep. The following conventional 3T MRI sequences were performed: (1) axial three‐dimensional (3D) inversion recovery fast spoiled gradient recalled echo T1‐weighted images (TE = 1.5 ms; TR = 6.3 ms; inversion time [TI] = 400 ms; flip angle, 15°) with 230 mm FOV, 156 contiguous partitions (1.0 mm) at 256 × 256 matrix; (2) axial T2‐weighted fluid‐attenuated inversion recovery images (TE = 126 ms; TR = 10 s; TI = 2200 ms) with 220 mm FOV, 47–48 contiguous slices (3.0 mm) at 256 × 256 matrix; and (3) axial magnetization‐prepared gradient echo T2*‐weighted d images (TE = 15 ms; TR = 500 ms; flip angle 20°) with 220 × 170 mm FOV and 47–48 contiguous slices (3.0 mm) at 256 × 192 matrix. Our main analysis included 265 participants who were scanned using a Siemens 3T TIM Trio scanner across seven different centers. We chose to analyze the data per scanner separately to reduce the effect of both within‐site and within‐scanner effects on the results commonly observed in other multi‐site and multi‐scanner studies (Dadar et al. 2020). This decision is supported by recent findings that highlighted the challenge of using MRI data from different manufacturers. Specifically, models trained on data from one manufacturer perform poorly when tested on data from another manufacturer. Additionally, the authors showed that harmonization using ComBat does not lead to noticeable improvements in classification performance across different scanner manufacturers (Kushol et al. 2023). Therefore, our approach ensures a more accurate comparison by directly addressing the inherent differences between scanners and sites. By analyzing the data per scanner, we can more reliably compare the results across different manufacturers and provide clearer insights into the effects observed. Hence, the Siemens scanner was chosen as it was the scanner with the highest number of mTBI cases (*n* = 216) and orthopedic controls (*n* = 49). Five participants were excluded from the main analysis due to the absence of either structural or functional files. Another two subsamples were used to replicate the analyses, a 3T General Electric (GE) Signa EXCITE scanner (mTBI cases *n* = 125; orthopedic controls *n* = 29 controls) and a 3T Phillips scanner (mTBI cases *n* = 149, orthopedic controls *n* = 17).

### 2.4 fMRI Preprocessing

Results included in this manuscript come from analyses performed using CONN (Whitfield‐Gabrieli and Nieto‐Castanon 2012) (RRID:SCR_009550) release 22.a (Nieto‐Castanon and Whitfield‐Gabrieli 2022) and SPM (Penny et al. 2011) (RRID:SCR_007037) release 12.7771. Functional and anatomical data were preprocessed using a flexible preprocessing pipeline (Nieto‐Castanon 2020) including realignment with correction of susceptibility distortion interactions, slice timing correction, outlier detection, direct segmentation and MNI‐space normalization, and smoothing. Functional data were realigned using the SPM realign and unwarp procedure (Andersson et al. 2001), where all scans were coregistered to a reference image (first scan of the first session) using a least squares approach and a 6‐parameter (rigid body) transformation (Friston et al. 1995), and resampled using b‐spline interpolation to correct for motion and magnetic susceptibility interactions. Temporal misalignment between different slices of the functional data (acquired in interleaved Siemens order) was corrected following the SPM slice‐timing correction (STC) procedure (Henson et al. n.d.; Sladky et al. 2011), using sync temporal interpolation to resample each slice BOLD timeseries to a common mid‐acquisition time. Potential outlier scans were identified using ART (Whitfield‐Gabrieli et al. 2011) as acquisitions with framewise displacement above 0.5 mm or global BOLD signal changes above three standard deviations (Nieto‐Castanon, unpublished manuscript; Power et al. 2014), and a reference BOLD image was computed for each subject by averaging all scans excluding outliers. Functional and anatomical data were normalized into standard MNI space, segmented into gray matter, white matter, and CSF tissue classes, and resampled to 2 mm isotropic voxels following a direct normalization procedure (Calhoun et al. 2017; Nieto‐Castanon, unpublished manuscript) using the SPM unified segmentation and normalization algorithm (Ashburner 2007; Ashburner and Friston 2005) with the default IXI‐549 tissue probability map template. Last, functional data were smoothed using spatial convolution with a Gaussian kernel of 8 mm full width half maximum (FWHM). In addition, functional data were denoised using a standard denoising pipeline (Nieto‐Castanon 2020) including the regression of potential confounding effects characterized by white matter timeseries (5 CompCor noise components), CSF timeseries (5 CompCor noise components), motion parameters and their first order derivatives (12 factors) (Friston et al. 1996), outlier scans (below 26 factors) (Power et al. 2014), session and task effects and their first order derivatives (2 factors), and linear trends (2 factors) within each functional run, followed by bandpass frequency filtering of the BOLD timeseries (Hallquist et al. 2013) between 0.008 Hz and 0.09 Hz. CompCor (Behzadi et al. 2007; Chai et al. 2012) noise components within white matter and CSF were estimated by computing the average BOLD signal as well as the largest principal components orthogonal to the BOLD average, motion parameters, and outlier scans within each subject's eroded segmentation masks. From the number of noise terms included in this denoising strategy, the effective degrees of freedom of the BOLD signal after denoising were estimated to range from 72.8 to 95.9 (average 84.6) across all subjects (Nieto‐Castanon, unpublished manuscript).

Functional connectivity multivariate pattern analyses (fc‐MVPA (Nieto‐Castanon 2022)) were performed to estimate the first 20 eigenpatterns characterizing the principal axes of heterogeneity in functional connectivity across subjects. From these eigenpatterns, 20 associated eigenpattern‐score images were derived for each individual subject characterizing their brain‐wide functional connectome state. Eigenpatterns and eigenpattern‐scores were computed separately for each individual seed voxel as the left‐ and right‐ singular vectors, respectively, from a singular value decomposition (group‐level SVD) of the matrix of functional connectivity values between this seed voxel and the rest of the brain (a matrix with one row per target voxel, and one column per subject). Individual functional connectivity values were computed from the matrices of bivariate correlation coefficients between the BOLD timeseries from each pair of voxels, estimated using a singular value decomposition of the *z*‐score normalized BOLD signal (subject‐level SVD) with 64 components separately for each subject (Whitfield‐Gabrieli and Nieto‐Castanon 2012). Group‐level analyses were performed using a General Linear Model (GLM (Nieto‐Castanon 2020). For each individual voxel a separate GLM was estimated, with first‐level connectivity measures at this voxel as dependent variables (one independent sample per subject and one measurement per task or experimental condition, if applicable), and groups or other subject‐level identifiers as independent variables. Voxel‐level hypotheses were evaluated using multivariate parametric statistics with random effects across subjects and sample covariance estimation across multiple measurements. Inferences were performed at the level of individual clusters (groups of contiguous voxels). Cluster‐level inferences were based on parametric statistics from Gaussian Random Field theory (Nieto‐Castanon 2020; Worsley et al. 1996). Results were thresholded using a combination of a cluster‐forming *p* < 0.001 voxel‐level threshold and a familywise corrected *p*‐FDR < 0.05 cluster‐size threshold (Chumbley et al. 2010). These analyses were performed for both fMRI timepoints. Additionally, a longitudinal analysis was conducted to examine changes in connectivity patterns over time, modeling the differences between the two timepoints. Post hoc analyses were conducted to further investigate the connectivity patterns of the brain regions associated with differences between mTBI cases and orthopedic controls identified by MVPA. Here, the MVPA clusters were taken as seeds in a seed‐to‐voxel analysis (voxel *p* < 0.001 and FWE cluster‐level *p* < 0.05 correction, *k* ≥ 40). Pearson's correlation coefficients were computed for the averaged time series within each MVPA cluster and the time series of all other voxels in the brain and were converted to normally distributed z‐scores using Fisher transformation prior to performing the second‐level general linear model. For all functional connectivity analyses, current age, sex, education, household income, site, and mean framewise displacement were included as covariates. MVPA has proved useful in uncovering unbiased differences in the functional connectome of various populations (Guell et al. 2020; Nieto‐Castanon 2022).

### 2.5 Neuropsychological Test Battery

Participants completed an in‐person neuropsychological battery conducted at 2 weeks, 6 months, and 1‐year post‐injury. The neuropsychological battery employed in the TRACK‐TBI study was designed to align with TBI Common Data Elements (CDEs) (Maas et al. 2010) and to target multiple cognitive domains frequently impacted by mTBI, such as executive function, visual‐motor skills, and memory (McInnes et al. 2017). The battery encompassed five scores, resulting from three different cognitive tests, that aimed to capture different domains of cognitive function. First, memory was assessed by the Rey Auditory Verbal Learning Test (RAVLT) (Rey 1958) including immediate recall score (sum of learning trials 1–5, score range 0–15 for each trial) and 20‐min delayed recall score (score range 0–15) (Rey 1958). Secondly, visual attention, task switching, and executive functioning were tested by the Trail Making Test (TMT) part A score (competition time, with a maximum of 100 s) (Reitan and Wolfson 1985) and part B score (competition time, with a maximum of 300 s) (Reitan and Wolfson 1985). Lastly, processing speed was assessed by the Processing Speed Index (PSI) from the Wechsler Adult Intelligence Scale–4th Edition (Wechsler 2019) (WAIS‐IV; a composite score reflecting performance on the Symbol Search subtest and the Coding subtest; age‐corrected mean 100, SD 15). These tests were chosen for their established sensitivity to cognitive deficits in TBI populations and their practicality in repeated assessments over time.

### 2.6 Statistical Analyses

All subsequent statistical analyses were performed in RStudio Version 1.7. *T*‐tests or Pearson's chi squared test of proportions were conducted to test for differences in demographic variables between scanners (see Table S1) and ANCOVAs controlling for age, sex and education to test for differences in cognitive tests with those with a mTBI and those without (Table 2). Model assumptions were checked using Q‐Q residual versus fitted plots to assume dichotomy of outcome variables. To test for an association between significantly different week 2 rs‐fMRI functional connectivity values extracted from post hoc analyses and cognitive assessments in the mTBI group, linear mixed‐effects models, with main effects of rs‐fMRI metric (extracting the biggest seed from each seed‐to‐voxel analysis), time (week 2, month 6, and month 12), and a rs‐fMRI metric*time interaction coupled with a participant‐specific random slope and intercept were performed separately for each cognitive outcome. False discovery rate (FDR)‐corrected simple slopes were tested post hoc to determine any marginal effects of the interaction term. FDR‐corrected *p* values after pooling all *p* values from each cognitive test for each model are presented. Significant influential outliers were checked in all models using Cooke's distance with a cutoff of 0.5. Education, age, biological sex, household income, site, and mean framewise displacement were accounted for in all models.

**TABLE 2:** TRACK‐TBI cognitive assessment (Siemens sub‐sample).

Cognitive tests	Timepoint	TBI (mean ± SD)	No TBI (mean ± SD)	*F*	df	*p*
Trail making test A	2 weeks	29.32 ± 13.50	27.48 ± 13.02	0.836	(1243)	0.362
	6 months	25.32 ± 9.46	24.55 ± 10.23	0.301	(1238)	0.584
	12 months	25.78 ± 13.17	20.67 ± 5.99	6.504	(1187)	0.011^a^
Trail making test B	2 weeks	73.09 ± 38.17	72.80 ± 40.91	0.003	(1243)	0.954
	6 months	66.16 ± 38.41	71.11 ± 42.27	0.958	(1236)	0.328
	12 months	67.60 ± 41.37	59.70 ± 22.76	1.546	(1186)	0.215
Wechsler Adult Intelligence Scale	2 weeks	9.49 ± 2.88	8.95 ± 2.91	1.556	(1241)	0.213
	6 months	10.37 ± 2.89	9.56 ± 3.28	3.288	(1239)	0.071
	12 months	10.69 ± 3.20	10.27 ± 3.67	0.666	(1184)	0.415
Rey auditory verbal learning test sum	2 weeks	45.45 ± 9.80	47.47 ± 9.99	1.861	(1243)	0.174
	6 months	46.19 ± 9.95	46.93 ± 12.16	0.193	(1239)	0.661
	12 months	48.65 ± 11.35	50.86 ± 11.20	1.527	(1187)	0.218
Rey auditory verbal learning test learnt	2 weeks	5.34 ± 2.27	5.45 ± 2.23	0.095	(1243)	0.758
	6 months	5.37 ± 2.09	5.55 ± 2.65	0.189	(1239)	0.664
	12 months	5.26 ± 2.04	5.35 ± 2.62	0.049	(1187)	0.824

Abbreviations: df, degrees of freedom; SD, standard deviation; TBI, traumatic brain injury.

^a^ Indicates significant effect at *p* < 0.05.

## 3 Results

A total of 265 participants aged 17–83 years with mTBI and orthopedic controls (*N* = 49) were included in the main analyses of the present paper under the criteria of being scanned by a single scanner manufacturer: Siemens. We replicate the initial findings using two different scanner manufacturers (GE, Phillips) where we also report the comparison of participant demographics between scanner types (see Table S1). Participant demographics and cognitive outcomes are found in Tables 1 and 2.

### 3.1 Differences in Functional Connectivity Between mTBI and Orthopedic Control Group at 2 Weeks Post‐Injury

**FIGURE 1:**
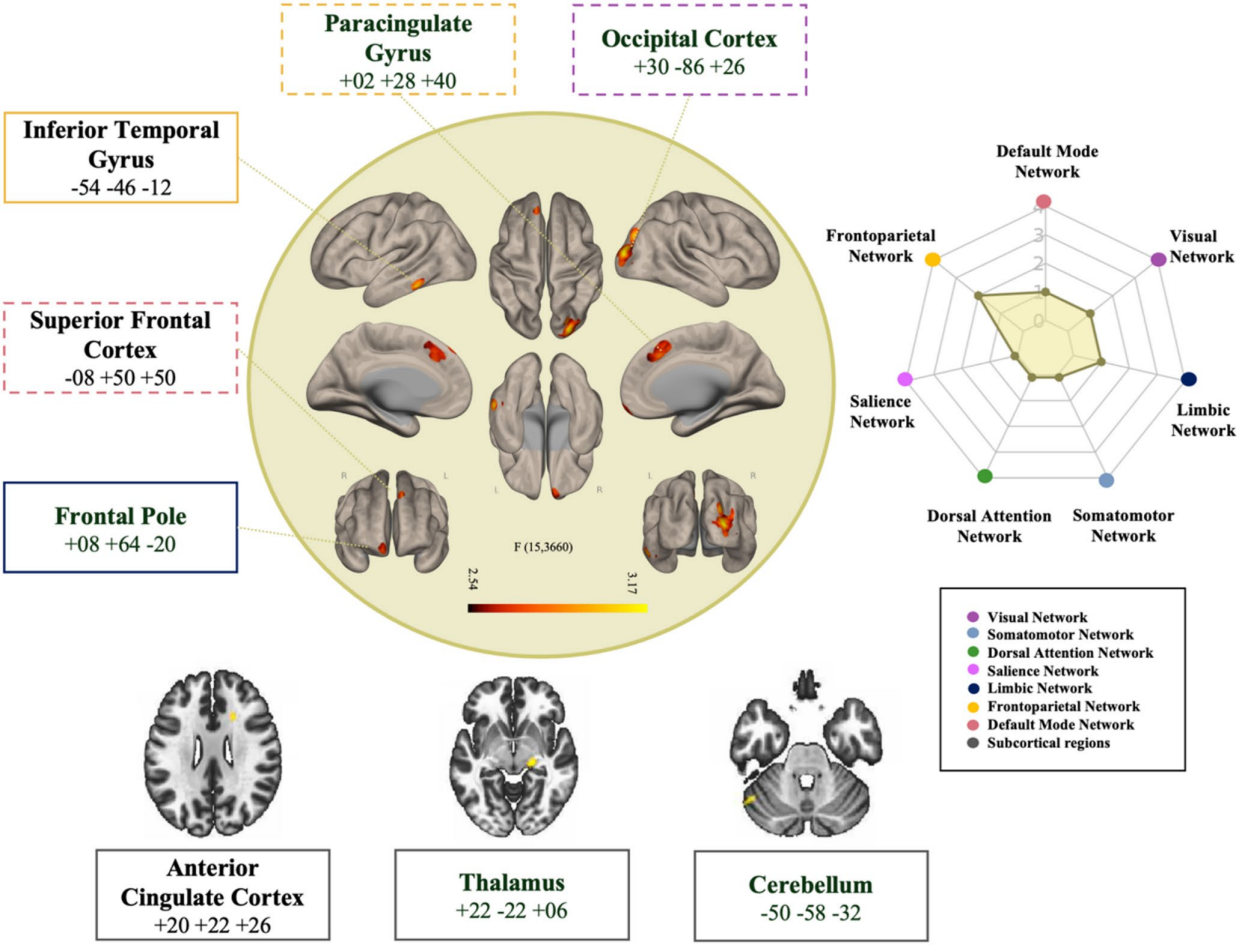
Voxel‐to‐voxel functional connectivity across the entire connectome revealed significant differences between mTBI and orthopedic controls in the functional connectivity patterns of eight significant clusters, including right occipital cortex, anterior cingulate gyrus, paracingulate gyrus, inferior and middle temporal gyrus, right thalamus, left cerebellum, and the bilateral frontal pole (including superior frontal cortex). On the right, the radar plot provides a visual summary of how the significant clusters are distributed across the seven intrinsic resting‐state networks defined by Yeo et al. (2011). Each plotted point represents a cluster, assigned to a specific network based on its MNI coordinates, as determined using FMRIB's Software Library (FSL) (Smith et al. 2004; Woolrich et al. 2009). The color coding follows the original Yeo network scheme, highlighting the spatial organization of the findings across functional networks. The dots map the anatomical seeds to their corresponding networks. The colors represent each of Yeo's 2011 networks, following the same pattern as the original framework. Two clusters were identified in the Frontoparietal Network (FPN), 1 cluster in the Default Mode Network (DMN), 1 cluster in the Limbic Network (LIM), and 1 cluster in the Visual Network (VIS). Dashed‐line text boxes highlight the replicated anatomical regions across scanners (see Supporting Information: Sections 3 and 4), emphasizing the stability and consistency of findings.

Voxel‐to‐voxel functional connectivity across the entire connectome revealed significant differences between TBI and controls in the functional connectivity patterns of eight significant (*p*‐voxel < 0.001, FEW cluster‐level *p* < 0.05) clusters (*k* > 40, Fmax = 15.36), including right occipital cortex, anterior cingulate gyrus, paracingulate gyrus, inferior and middle temporal gyrus, right thalamus, left cerebellum, and the bilateral frontal pole (including superior frontal cortex) (Figure 1, full model in Table S2). Replication analyses using General Electric (GE) (*n* = 155) and Phillips (*n* = 166) scanner samples yielded both consistent and scanner‐specific findings. The GE analysis revealed five clusters, including the paracingulate/superior frontal gyrus, lateral occipital cortex, hippocampus, occipital pole, and supplementary motor area, while the Phillips sample identified eight clusters across regions such as the precuneus, middle temporal gyrus, superior frontal gyrus, frontal pole, pars triangularis, and frontal orbital cortex. Importantly, anatomical areas overlapping across replicates included the paracingulate gyrus, occipital cortex, and superior frontal gyrus. These overlapping areas included regions within the default mode network (DMN), frontoparietal network (FPN), and visual network (VIS), underscoring the stability and meaningfulness of these findings across datasets (see Figure 1, and Supporting Information for detailed replication results, Figures S1 and S2, and Tables S3 and S4).

**FIGURE 2:**
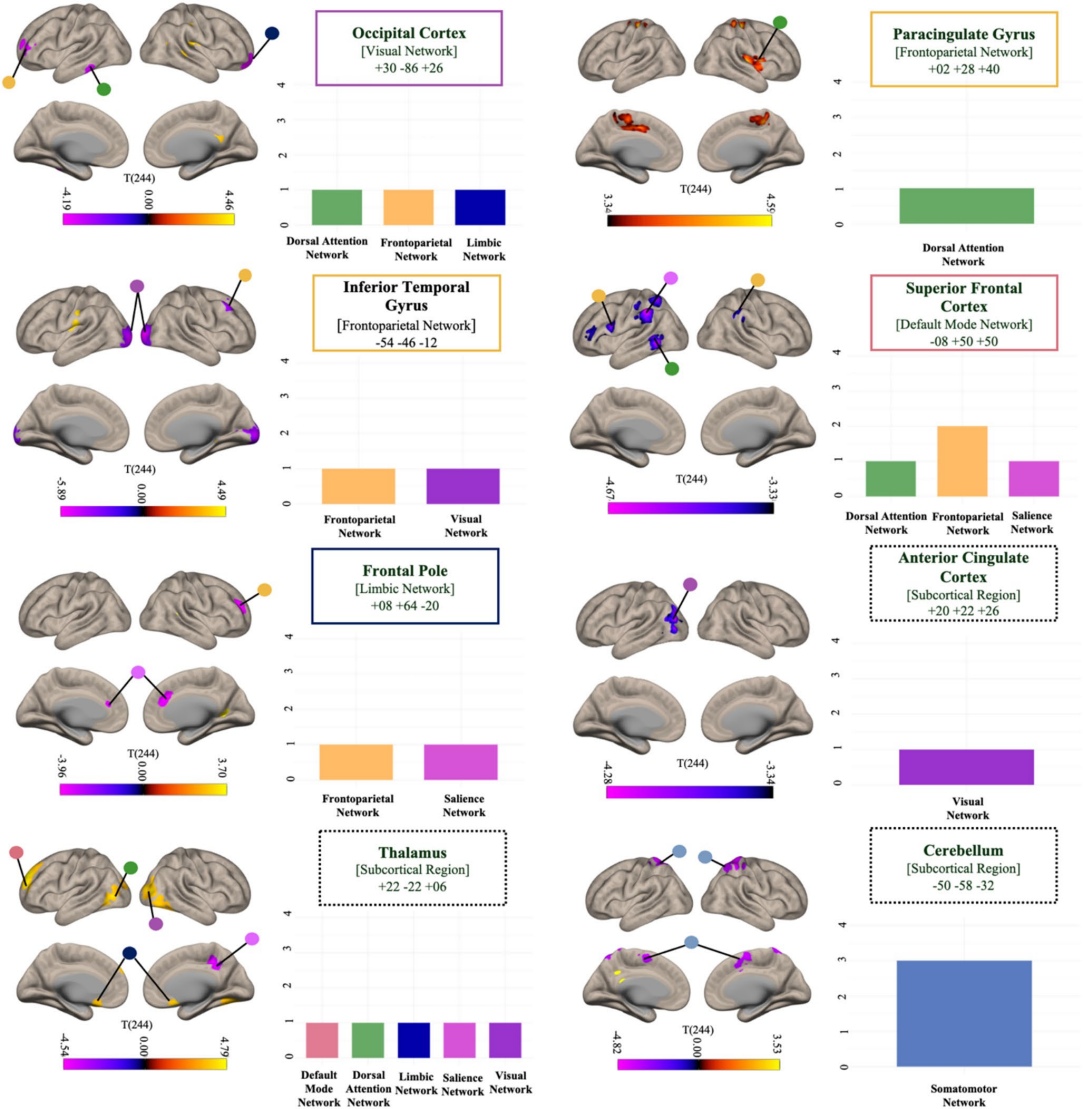
Post hoc seed‐to‐voxel results for each significant MVPA cluster. The bar plot provides a visual summary of how the significant clusters are distributed across the seven intrinsic resting‐state networks defined by Yeo et al. (2011), with the *x*‐axis representing each network name and the *y*‐axis showing the number of significant clusters. The color coding follows the original Yeo network scheme. Each cluster was assigned to a specific network based on its MNI coordinates, as determined using FMRIB's Software Library (FSL) (Smith et al. 2004; Woolrich et al. 2009). The color bar below the brain maps represents the directionality of the difference, with anticorrelations (or hypoconnectivity relative to the control group) shown in violet to black and hyperconnectivity (relative to the control group) from black to yellow. Patterns of functional connectivity differences in mTBI versus orthopedic controls are driven by a mix of hyper and hypoconnectivity within and between large‐scale intrinsic functional networks, particularly within and between the FPN and DMN.

Post hoc characterization of each significant cluster revealed by MVPA using seed‐to‐voxel analysis revealed multiple areas of cerebral cortical hyperconnectivity and hypoconnectivity in mTBI when compared with the control group. We report the network overlap of each post hoc cluster in Figure 2 and full model in Table 3.

**TABLE 3:** *Post hoc* full models. FDR, false discovery rate.

MVPA seed ROI			Seed‐to‐voxel cluster result		
Label	Mean MNI coordinate	Label	Mean MNI coordinate	Size	Size *p*‐FDR
Occipital Cortex	+30 – 86 + 26	Right Thalamus	+34 –18 +30	3117	0.000000
		Frontal Pole Left	−32 +56 +8	350	0.025288
		Inferior Temporal Gyrus	−46 –40 –16	311	0.027893
		Frontal Pole Right	+20 + 56 –14	250	0.048085
Paracingulate Cortex	+02 +28 +40	Precentral Gyrus	+16 –32 +58	1787	0.000000
		Insular Cortex	+30 –16 +16	547	0.003002
		Central Opercular Cortex	+50 +02 +04	483	0.004065
Inferior Temporal Cortex	−54 –46 –12	Occipital Pole	+26 –94 +02	3531	0.000000
		Brain‐Stem	+04 –28 –20	466	0.002482
		Central Opercular Cortex	−42 –20 26	447	0.002482
		Middle Frontal Gyrus	+38 +26 +50	420	0.002482
		Cerebellum	+32 –62 –36	408	0.002482
Thalamus	+22 –22 –06	Occipital Cortex Right	+38 –76 +16	2321	0.000000
		Superior Frontal Gyrus	−18 +54 +36	1030	0.000008
		Bilateral Thalamus	+08 –22 +18	581	0.000530
		Occipital Cortex Left	−42 –66 +04	564	0.000530
		Cingulate/Precuneus	+16 –38 +40	492	0.001003
		Subcallosal Cortex	+04 +18 –16	237	0.027186
Anterior Cingulate	+20 +22 +26	Occipital Cortex	−44 –78 +18	856	0.000044
		Hypothalamus	+20 –38 +28	468	0.002077
Frontal Pole	+08 +64 –20	Anterior Cingulate	+14 +28 +32	322	0.025962
		Frontal Pole Right	+26 +52 +18	296	0.025962
		Precuneus Cortex	+36 –46 +20	292	0.025962
Cerebellum	−50 –58 –32	Superior Parietal Right	+30 –44 +66	1342	0.000001
		Hypothalamus	−20 –46 +18	649	0.000354
		Superior Parietal Left	−26 –44 +70	563	0.000627
		Supplementary Motor Cortex	+08 –02 +64	368	0.005324
Superior Frontal Cortex	−08 +50 +50	Left Supramarginal and Superior Parietal Lobule	−60 –36 +42	1443	0.000000
		Middle Temporal Gyrus	−60 –58 –02	497	0.002614
		Supramarginal/Postcentral Gyrus	+50 –34 +38	293	0.025843
		Inferior Frontal/Precentral Gyrus	−48 +08 +20	225	0.046324
		Inferior Frontal Gyrus	−40 +30 +08	221	0.046324

### 3.2 Differences in Functional Connectivity Over Time (2 Weeks Versus 6 Months) Between mTBI and Orthopedic Control Group

Voxel‐to‐voxel functional connectivity across the entire connectome revealed no significant group‐level differences between the mTBI and orthopedic control groups in functional connectivity pattern changes from 2 weeks to 6 months.

### 3.3 Correlation Between 2‐Week Functional Connectivity and Changes in Cognition Over Time

To test if changes in cognition over 12 months were a function of differences in week 2 functional connectivity in the mTBI group (extracting and testing the biggest seed from each seed‐to‐voxel *post hoc* analysis), linear mixed‐effects models with week 2 rs‐fMRI metrics and time (week 2, month 6, and month 12) revealed a significant time*functional connectivity interaction with functional connectivity between the right frontal pole and anterior cingulate gyrus and TMT‐B scores (*β* = −25.90, *p* value FDR corrected = 0.020, CI = −43.526, −8.282, *Conditional R* ^2^ = 0.774). Additionally, a significant time*functional connectivity interaction was also observed in the connectivity between inferior temporal and bilateral occipital pole (*β* = −12.26, *p* value FDR corrected = 0.008, CI = −20.243, −4.291, *Conditional R* ^2^ = 0.675) and in the connectivity between cerebellum and superior parietal pole (*β* = 13.98, *p* value FDR corrected = 0.002, CI = 5.676, −22.295, *Conditional R* ^2^ = 0.677) and TMT‐A scores. We did not find a significant time*functional connectivity interaction with the TMT B‐A, RAVLT, and WAIS assessments (see full model results in Supporting Information, Table S5).

**FIGURE 3:**
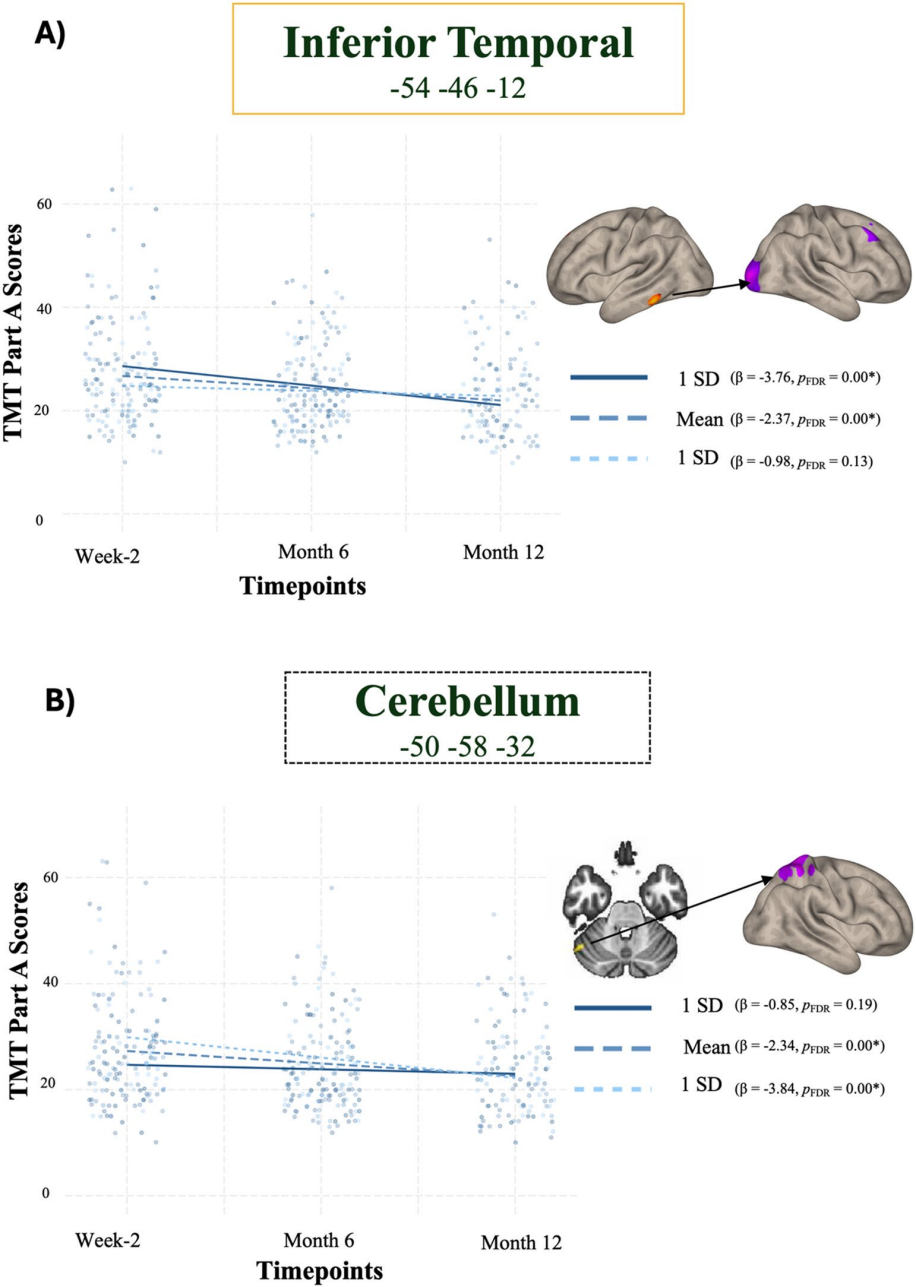
Line plot of the interaction between time and FC illustrates that the change in TMT‐A over 12 months is different at different levels of functional connectivity between the Inferior temporal pole (Frontoparietal Network) and the bilateral occipital pole (Visual Network) (Panel A) and the Cerebellum and the superior parietal pole (Somatomotor Network) (Panel B). Dashed lines represent the group mean FC *z*‐score, the +1SD FC z‐score is represented by the solid lines, and the −1SD is represented by the dotted lines. FDR, false discovery rate; SD, standard deviation; TMT, trail making test.

Post hoc marginal effects analysis, taking the inferior temporal and bilateral occipital pole connection at its mean, and 1 standard deviation (SD) above the mean, revealed negative associations with TMT‐A over time (*β* = −2.37, *p*_FDR_ = 0.00) and +1SD (*β* = −3.76, *p*_FDR_ = 0.00), whereas—1SD below the mean (*β* = −0.98, *p*_FDR_ = 0.13) was not significantly associated with better scores in TMT‐A over time. Equally, functional connectivity between the cerebellum and superior parietal pole at its mean, and 1 standard deviation (SD) below the mean, revealed negative associations with TMT‐A over time (*β* = −2.34, *p*_FDR_ = 0.00) and −1SD (*β* = −3.84, *p*_FDR_ = 0.00), whereas +1SD above the mean (*β* = −0.85, *p*_FDR_ = 0.19) was not significantly associated with better scores in TMT‐A over time (Figure 3). Additionally, functional connectivity between the anterior cingulate cortex and bilateral occipital pole at 1 standard deviation (SD) above the mean revealed a significant negative association with TMT B‐A score over time (*β* = −2.37, *p*_FDR_ = 0.00). However, at the mean connectivity level (*β* = −1.30, *p*_FDR_ = 0.24) and −1 SD below the mean (*β* = 2.20, *p*_FDR_ = 0.16), the associations with TMT B‐A scores over time were not statistically significant.

**FIGURE 4:**
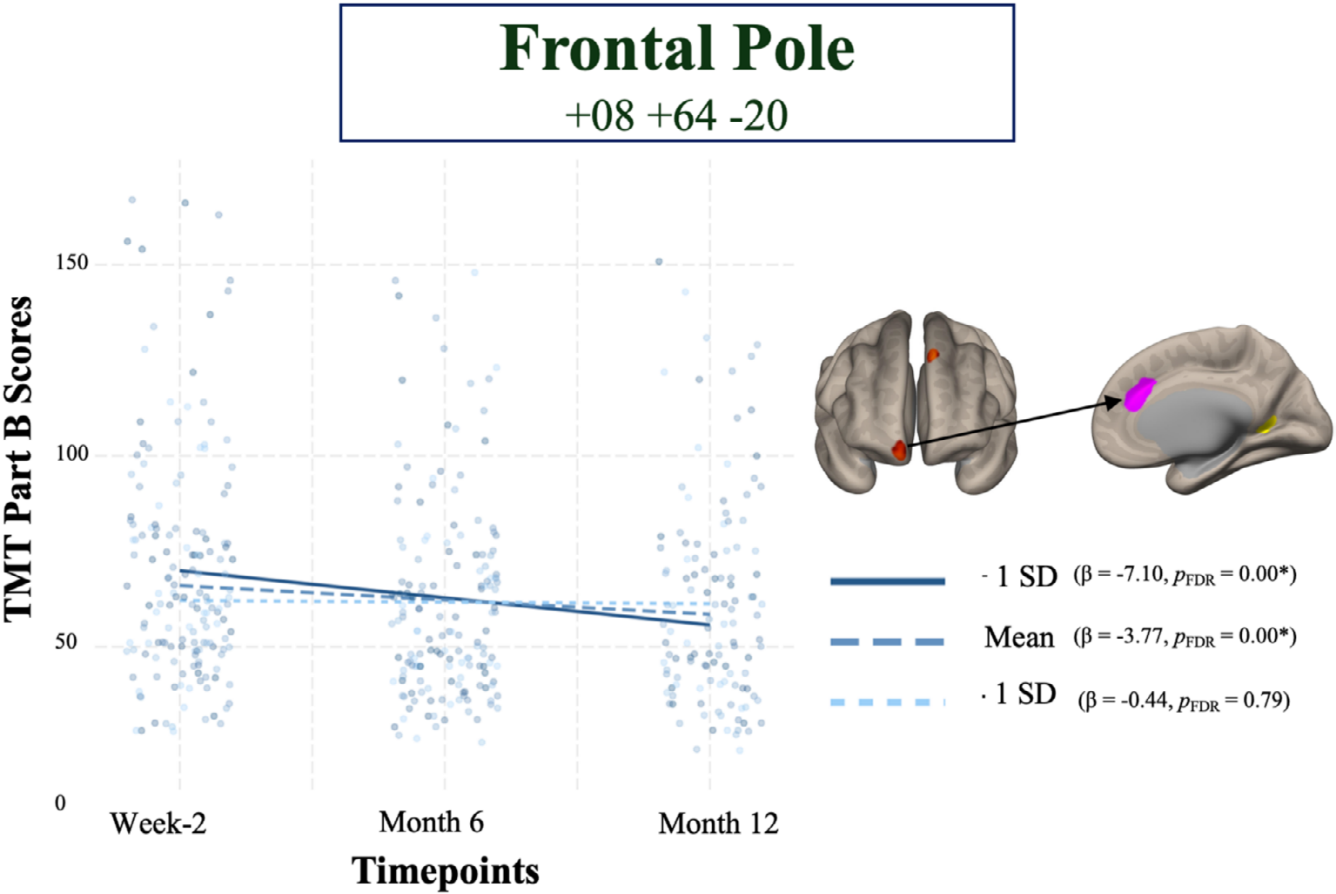
Line plot of the interaction between time and FC illustrates that the change in TMT‐B over 12 months is different at different levels of functional connectivity between Frontal pole (Limbic Network) to anterior cingulate gyrus (Salience Network). Less anti‐correlation at two weeks post injury between these two regions (+1SD FC *z*‐score above the mean) was associated with a greater improvement in TMT‐B. Dashed lines represent the group mean FC *z*‐score, the +1SD FC *z*‐score is represented by the solid lines, and the −1SD is represented by the dotted lines. FDR, false discovery rate; SD, standard deviation; TMT, trail making test.

For TMT‐B, post hoc marginal effects analysis taking functional connectivity between the right frontal pole and anterior cingulate gyrus at its mean, and 1 standard deviation (SD) above the mean, revealed negative associations with TMT‐B over time (*β* = −3.77, *p*_FDR_ = 0.00) and +1SD (*β* = −7.10, *p*_FDR_ = 0.00), whereas −1SD below the mean (*β* = −0.44, *p*_FDR_ = 0.79) was not significantly associated with better scores in TMT‐B over time (Figure 4).

## 4 Discussion

This study aimed to analyze whole‐brain functional connectivity patterns in a large, well‐characterized sample to distinguish sub‐acute mTBI cases from a matched group of orthopedic control subjects. Using data‐driven unbiased fc‐MVPA analysis, we compared connectivity across the entire connectome between the two groups. While no longitudinal changes were observed between the 2‐week and 6‐month timepoints, our main voxel‐to‐voxel analysis at the 2‐week post‐injury revealed significant differences in connectivity patterns across eight clusters in individuals with sub‐acute mTBI compared to orthopedic controls. Differences in connectivity patterns were seen in the right occipital cortex, anterior cingulate gyrus, paracingulate gyrus, inferior and middle temporal gyrus, right thalamus, left cerebellum, and bilateral frontal pole (encompassing the superior frontal cortex). Consistent with previous reports (Li et al. 2020) these differences were predominantly located within regions associated with crucial networks in the recovery process after mTBI: FPN, DMN, as well as several other regions pertaining to the VIS and LIM. The secondary aim of our study was to examine whether specific functional connectivity patterns obtained in post hoc analyses are linked to cognitive performance at 2 weeks, 6 months, and 12 months post‐mTBI. Higher positive connectivity (hyperconnectivity compared to controls) between the inferior temporal gyrus and bilateral occipital gyrus was associated with greater improvement in visuo‐motor skills. Similarly, higher positive connectivity (hyperconnectivity compared to controls) between the right frontal pole and the anterior cingulate cortex was associated with greater improvement in executive set shifting abilities over 12 months. Conversely, greater anticorrelations (hypoconnectivity compared to controls) between the cerebellum and the parietal lobe were associated with greater improvement in visuo‐motor skills over 12 months.

### 4.1 Functional Connectivity Differences Between Sub‐Acute mTBI and Orthopedic Controls

Because fc‐MVPA is an omnibus test, post hoc seed‐to‐voxel analyses are performed to better characterize which connections are driving differences in functional connectivity patterns between groups. Here we draw conclusions from a network perspective with emphasis on those network seeds that replicated across scanner types: the DMN, FPN, and VIS. The overlap of these networks across independent datasets underscores the robustness and stability of our findings, despite scanner‐related variability. These results highlight the critical involvement of these networks in the pathophysiology of mTBI (see Supporting Information for detailed replication results, Figures S1 and S2, and Tables S3 and S4). Firstly, patterns of functional connectivity of the cluster within the DMN (namely the superior frontal cortex, seen in the main analyses and the Phillips scanner group) exhibited increased anticorrelations, or hypoconnectivity, with two regions of the FPN, as well as the SAL, DAN, and ipsilateral sensory association areas compared to controls. This consistent pattern of DMN disconnection aligns with previous studies showing reduced activation in the DMN in individuals with mTBI (Abdul Rahman et al. 2022; Bai et al. 2023; Johnson et al. 2012; Mayer et al. 2011; Zhou et al. 2012). The DMN reduced connectivity has been correlated with greater post‐concussion symptoms (Dean et al. 2015b; Palacios et al. 2017). However, our data diverge from the findings of van der Horn et al. (2017) who reported patterns of hyperconnectivity in the DMN and persistent complaints at 3 months post‐injury in mTBI. This discrepancy may be attributable to the fact that the significant results in the van der Horn et al. study were confined to the anterior and posterior regions of the DMN, rather than reflecting connectivity with the rest of the brain. Additionally, participants in this current analysis were scanned within more acute phases of mTBI recovery (< 2 weeks since injury), potentially reflecting temporal fluctuations in DMN connectivity over time. Secondly, clusters within the FPN (namely the cingulate clusters, seen in the main analysis and in the GE scanner group) exhibited a consistent pattern of hyperactivity with multiple regions and networks. Our results align with previous literature that showed in comparison to the controls, an increase of FPN connectivity (Shumskaya et al. 2012) and preserved global network characteristics are seen after mTBI (Kim et al. 2022). Conversely, this finding is inconsistent with previous studies that report less efficient network communication in individuals with mTBI, especially in those with post‐concussion syndrome, compared to controls (Hou et al. 2019; Zhou 2017). Again, discrepancies in results across studies could be reflective of a temporal time course of network reorganization after mTBI, which highlights the importance of imaging participants at consistent times since injury. Lastly, the occipital cortex, consistent across all three scanner replication analyses, exhibited long‐range disconnections between VIS and temporal hubs of the DAN and frontal regions of the FPN and LIM. Our results align with extensive literature indicating that impaired visual information processing is a hallmark of mTBI linked to disconnections of the VIS with the rest of the functional networks correlated with cognitive dysfunctions in sub‐acute mTBI (Li et al. 2020; Ruiz et al. 2024). However, we also found that the VN showed a pattern of hyperconnections with subcortical regions such as the thalamus. Given the thalamus's role in integrating visual information, this connectivity change after mTBI could suggest a potential compensatory mechanism or heightened cortical–subcortical processing, indicating the brain's adaptive response to preserve visual processing integrity despite the susceptibility of long‐range connections to persistent axonal swelling and disconnection typically observed in TBI (Mckee and Daneshvar 2015).

An important consideration in interpreting functional connectivity changes following mTBI is the role of time since injury. In this study, we focused on the early subacute phase, specifically between 5 and 18 days post‐injury, which provides valuable insights into the early recovery trajectory. However, the distinction between “acute” and “sub‐acute” stages in the literature can sometimes be blurred, and in our manuscript, we acknowledge the need for clearer delineation of these phases. Functional connectivity in mTBI is likely to evolve over time, with different patterns emerging at various stages of recovery (D'Souza et al. 2020; Morelli et al. 2021). In the acute phase, connectivity disruptions dominate due to injury and inflammation, manifesting as widespread reductions across key behavioral networks. In the subacute phase, a complex interplay of maladaptive changes and recovery‐driven neuroplasticity emerges. This includes persistent disconnections in networks such as the DMN and FPN, alongside localized hyperconnectivity in regions associated with network organization and mitigating network disruptions like the posterior cingulate cortex and precuneus (D'Souza et al. 2020; Iraji et al. 2015; Mayer et al. 2015; Murdaugh et al. 2018; Palacios et al. 2017; Vakhtin et al. 2013; van der Horn et al. 2017; Zhou et al. 2012). The subacute phase also shows evidence of anterior–posterior network differences, with reduced connectivity in anterior regions (e.g., anterior DMN and FPN) and increased connectivity in posterior regions. The cerebellar network, in particular, exhibits consistent hyperconnectivity during this phase, potentially reducing the burden on cortically driven executive resources to support motor and cognitive demands (Manning et al. 2017; Murdaugh et al. 2018; Nathan et al. 2015; Vergara et al. 2017). By the chronic phase, connectivity patterns stabilize, reflecting either recovery or chronic dysfunction (D'Souza et al. 2020). Persistent DMN disconnection or anterior hypoconnectivity, coupled with maladaptive hyperconnectivity in networks such as the FPN, may underlie long‐term impairments. Hyperconnectivity within the cerebellum remains prominent, likely reflecting a compensatory mechanism to maintain motor and cognitive functions (D'Souza et al. 2020; Morelli et al. 2021). A recent study expands on prior evidence by demonstrating that increases in functional connectivity can be observed as late as 18 months post‐injury. Critically, the age of the patient at the time of injury influenced the extent of these changes. Specifically, younger adults showed more extensive increases in both intra‐ and inter‐network resting‐state functional connectivity compared to older adults. These stabilization patterns, including persistent anterior–posterior imbalances, highlight the enduring complexity of functional connectivity responses to mTBI. While our findings highlight connectivity alterations in the sub‐acute period, future research should further explore how these changes develop over the longer term. Longitudinal studies that span the acute, sub‐acute, and chronic stages of recovery would allow for a more comprehensive understanding of both adaptive and maladaptive neural changes. Understanding these network dynamics could provide critical insights into the persistence of functional impairments or compensatory mechanisms, particularly in individuals who do not fully recover. Examining connectivity patterns in chronic stages may also identify markers for intervention, offering new avenues for targeting persistent dysfunction or promoting late‐stage recovery.

In summary, the diverse connectivity patterns highlighted at 2 weeks post‐injury underscore the complex neurobiological changes following mTBI. Specifically, our results highlighted a distinct reorganization of brain networks in sub‐acute stages of mTBI characterized by a global disconnection of the DMN from the rest of the brain and a concomitant FPN hyperconnectivity. Many studies discussed here utilized varied analytical methods, including seed‐based, ROI‐based, and ICA methodologies. By employing a data‐driven, unbiased approach, our study offers a novel perspective in the literature, providing more precise insights into the connectivity alterations and network reorganization of the DMN, FPN, and VIS following mTBI.

### 4.2 Functional Connectivity Patterns and Cognitive Performance Over Time in mTBI

The secondary aim of our study was to examine whether specific functional connectivity patterns obtained in post hoc characterization of the unbiased fc‐MVPA clusters are linked to cognitive performance at 2 weeks, 6 months, and 12 months post‐mTBI. Visual‐motor skills assessed with TMT‐A over 12 months post injury were significantly associated with FPN‐VIS network anticorrelations, along with decreased anticorrelations between the cerebellum and extensive areas of the SMN. Additionally, these specific patterns of connectivity at or above the mean (FPN‐VIS) and at or below the mean (cerebellum—SMN) were associated with significantly maintained visual‐motor skills over time. The results are in line with previous literature reporting that strength of resting state functional connectivity in identified visual‐frontal networks associated with mTBI may be neural mechanisms that underlie neurocognitive abilities (Gilmore et al. 2016). Previous reports have also focused on motor networks, highlighting the role of the SMN in motor learning and suggesting its potential involvement in disease‐related alterations in functional connectivity during motor tasks (Kumar et al. 2018). A similar conclusion was previously reached where post‐injury coactivity within the cerebellar and SMN was strongly associated with reaction time during cognitive testing (Nathan et al. 2015). Additionally, decreased functional connectivity within the motor‐striatal network has been reported in mTBI (Shumskaya et al. 2012). Conversely, a recent study examining sex differences in abnormal intrinsic functional connectivity after acute mTBI showed that increased SMN and cerebellum within connectivity were reported but just in male patients compared to either male healthy controls or female patients (Wang et al. 2018). While much research has focused on within‐network connectivity of the SMN and cerebellar network, we bring further clarity by identifying the most prominent hubs involved in the interplay of these two networks. Overall, our findings underscored the critical role of brain connectivity interactions, where increased FPN‐VIS connectivity and maintained cerebellum‐SMN dynamics appeared crucial in predicting and potentially influencing long‐term visual‐motor cognitive outcomes following mTBI.

Similar to the FPN‐VIS network and TMT‐A associations, those whose connectivity between the right frontal pole (LIM) and the anterior cingulate cortex (SAL) was positive had greater improvement in TMT‐B over time. While these results possibly add to our understanding of specific cortical connections responsible for cognitive deficits post mTBI, our analyses demonstrate the high heterogeneity of cortical reorganization post‐injury and how such heterogeneity is important for predicting outcomes. Increased connectivity in some individuals could reflect either compensatory mechanisms trying to maintain function or hyperexcitability as a result of underlying brain damage. Evidence for the latter is seen here, where those with hyperconnectivity in this pathway showed worse baseline scores but a steeper improvement slope, suggesting that this hyperexcitability could potentially reflect early damage and a greater potential for recovery. Conversely, those demonstrating increased anticorrelations in this edge might have shown better initial scores in cognitive performance with minimal subsequent change. This pattern suggests a different adaptive response, possibly involving either a compensatory mechanism or the preservation of normal anticorrelation patterns typically seen in healthy individuals (Li et al. 2021). Our findings highlighted the heterogeneity of neural connectivity responses following injury, suggesting that variations in initial impairment levels may influence subsequent connectivity patterns. In line with previous research, our results underscored the complex interplay between neurological resilience, compensatory mechanisms, and the progression of TBI physiopathology (Morelli et al. 2021). Questions remain regarding adaptive or maladaptive connectivity changes after mTBI, and further studies are warranted to elucidate the interindividual heterogeneity and the temporal time course of connectivity changes after mTBI.

While the role of anti‐correlations in rs‐fMRI remains debated, there is increasing evidence suggesting that these negative correlations reflect a genuine biological process and an important aspect of brain network organization (Chai et al. 2012; Fox et al. 2009; Keller et al. 2013; Li et al. 2021). Specifically, anti‐correlations between networks, such as the DMN and task‐positive regions, have been linked to better cognitive task performance and better severity scores in clinical disorders including schizophrenia (Whitfield‐Gabrieli et al. 2009), attention‐deficit/hyperactivity disorder (Castellanos et al. 2008), bipolar disorder (Chai et al. 2011) and Alzheimer's disease (Wang et al. 2007). Increased anti‐correlations have also been predictive of depressive symptom improvement following an intervention (Fox et al. 2014; Weigand et al. 2018). In the context of TBI, however, widespread white matter lesions and/or focal gray matter damage are likely to lead to disconnection of long‐range functional networks and disruptions in local processing, which could theoretically lead to structural disconnection and hypoconnectivity within and between cortical regions and functional networks that would otherwise be positively connected. Such disconnection might be manifested as anti‐correlations in rs‐fMRI data. Conversely, the same disconnection/damage could also theoretically lead to increased connectivity as a compensatory mechanism to maintain function, despite anatomical damage, manifesting as hyperconnectivity when compared to an uninjured group. Indeed, the variability in seed‐to‐cluster pairs seen in our analysis was related to changes in TMT performance over time. Those with +1SD FC z‐scores above the mean between the frontal pole and the anterior cingulate cortex showed significantly greater improvements in TMT performance over 12 months. Those in this +1SD group showed positive connectivity between these two regions (see Figure S3), which is indicative of a normal connectivity profile in healthy persons (Fettes et al. 2018). This could be interpreted in one of two ways: either these participants resulted in less damage because of their injury, and their improvement was a learning effect, or this connectivity profile reflects a compensatory mechanism, minimizing the loss of connectivity, which is seen in those with mean and −1SD below the mean FC *z*‐scores (see Figure S3). Importantly, those whose connectivity between the frontal pole and the anterior cingulate was negative (i.e., anti‐correlated) displayed less improvement in TMT performance over time. Similarly, this was the case for the inferior temporal (frontoparietal network) and occipital cortex (salience network) clusters also. As such, anti‐correlated connections between regions that typically show positive connectivity (specifically to regions within the salience network) following mTBI may hold prognostic promise in understanding cognitive recovery.

Overall, these findings underscore the importance of understanding interindividual differences in connectivity patterns post‐injury, as they could hold critical implications for clinical practice. For example, the observed connectivity patterns could inform early screening protocols by identifying imaging markers indicative of potential recovery or persistent deficits. Screening tools could integrate these connectivity markers to stratify patients based on their recovery potential, enabling tailored monitoring and interventions. From a prognostic perspective, altered connectivity patterns, such as those seen in the FPN‐VIS and cerebellum‐SMN interactions, might provide insight into the likelihood of cognitive recovery or the persistence of deficits. Patients with preserved or adaptive connectivity dynamics could potentially benefit from less intensive interventions, while those with maladaptive patterns might require more targeted therapeutic strategies. Finally, these findings have implications for intervention design. Therapeutic strategies could aim to promote adaptive connectivity changes, for instance, by leveraging neurostimulation techniques, cognitive rehabilitation programs, or task‐specific motor training to enhance network dynamics. Furthermore, sex‐specific interventions might be warranted, given prior evidence of differential connectivity patterns between males and females post‐mTBI (Wang et al. 2018) and our preliminary results (see Table S6, Figure S4–S8).

Our results not only expand our understanding of neural mechanisms underlying cognitive outcomes in mTBI but also highlight the potential for connectivity patterns to guide personalized care. Future longitudinal studies are essential to explore how these findings translate into clinical practice, particularly in refining screening, prognostic modeling, and intervention strategies for mTBI patients.

### 4.3 Limitations

Our results should be interpreted considering several limitations. Firstly, the main analysis was cross‐sectional and did not include pre‐injury data, despite the recognition that pre‐injury status is likely to have a substantial impact on the recovery trajectory of individuals with mTBI (Yue et al. 2019). Secondly, in our study, significant findings were observed with TMT‐A and TMT‐B, yet no statistically significant differences were noted with RAVLT and WAIS assessments. This finding may be attributed to a reliance on isolated cognitive tests for each domain rather than a comprehensive cognitive battery, potentially limiting the ability to fully capture the spectrum of cognitive deficits following mTBI. Even though, our findings align with existing literature indicating that executive function impairments are a hallmark outcome of mTBI (Ozga et al. 2018). However, while broader assessments of memory or additional cognitive domains, such as visuospatial memory or semantic processing, could provide a more comprehensive understanding, their exclusion was due to protocol design constraints that prioritized feasibility and minimized participant burden. Importantly, the selected tests offer robust measures of the core cognitive functions most affected by TBI, enabling the examination of key associations between injury and recovery. The choice of cognitive tests inherently shapes the observed associations, as different assessments may emphasize distinct aspects of cognition. Future research may build on this work by incorporating broader cognitive measures to explore their relationships with outcomes in TBI populations, potentially uncovering additional patterns of cognitive resilience or impairment. In fact, identifying enduring cognitive domains affected in chronic‐stage mTBI remains unclear (Ponsford et al. 2012). Thirdly, while our study has accounted for age and sex in all analyses, it is crucial to acknowledge findings from other studies indicating significant differences in recovery and connectivity following mTBI across the lifespan and in different sexes (Levin et al. 2021). Interestingly, recent research highlights that older age at injury and male sex are risk factors for post‐injury network degradation (Amgalan et al. 2022). In our exploratory analysis, conducted within the Siemens subsample (see Table S6, Figure S4–S8), we observed sex‐specific differences in connectivity patterns, particularly in regions such as the lateral occipital cortex, paracingulate gyrus, thalamus, and inferior/middle temporal gyrus. These promising findings suggest that sex‐based variability in mTBI‐related connectivity alterations may play an important role in shaping recovery trajectories, offering valuable insights for future studies. Given the growing recognition of sex as a critical biological variable impacting injury outcomes and treatment efficacy (Gupte et al. 2019), these initial findings highlight the need for more focused investigations into sex differences and sex‐specific factors in the context of mTBI. Future research should aim to explore these connectivity patterns in greater depth, with sex‐specific analyses that can better inform how these differences influence recovery and brain function in both males and females. Larger, stratified samples will be essential to fully understand these patterns and to refine targeted interventions that address the unique needs of each sex in the recovery process. Fourth, our primary strength lies in utilizing a data‐driven approach with multivariate pattern analysis to explore unbiased whole‐brain rsFC changes associated with mTBI. Nonetheless, this method has its limitations. While MVPA reduces input features, potentially mitigating false correlations (McNorgan et al. 2020) any data‐driven approach inherently increases the risk of modeling by chance. Therefore, inferences should be drawn from the initial MVPA model, rather than from post hoc analyses, as these can introduce bias and distort the true relationships between variables (Nieto‐Castanon 2022). To strengthen the reliability of our findings, we employed replication across two independent datasets, ensuring that observed patterns are generalizable and not specific to a single dataset. This approach also helps address site and scanner effects, reducing the potential confounding influence of these technical factors (Kushol et al. 2023). Focusing on results that replicate across the three datasets reduces the risk of overfitting in a similar way to cross‐validation by testing the generalizability of our findings across different data sources. Although the initial MVPA from which we make statistical inferences does not provide direct insight into the specific neural mechanisms behind the observed patterns, post hoc seed‐to‐voxel analyses provide improved interpretability and a basis for future research in independent datasets and strategies like replication, careful interpretation of the regions or networks identified, and cautious examination of effect sizes help improve the clarity and reliability of the findings. By incorporating these methods, we aim to reduce overfitting, enhance interpretability, and ensure the robustness of our MVPA results. Fifth, since the study has been conducted across three different samples defined by scanner manufacturer, ANOVAs were conducted to evaluate differences in demographic variables across each sample. The analyses revealed significant differences in age and education levels among the three scanners. No significant difference was observed for household income. Additionally, a chi‐squared test indicated no significant difference in sex distribution across scanner types (see Table S1). These findings suggest that household income and sex distribution are similar across scanner types, ensuring that any observed differences in the results are less likely to be influenced by these demographic factors. Adjusting these factors in our models helps mitigate their potential impact, but we believe that differences across scanners justify our choice to analyze these data separately. Sixth, while the WAIS PSI composite score was used to assess processing speed, the individual contributions of the visual search and working memory subtests were not explored. The WAIS PSI score, as a composite measure, incorporates both age‐corrected scaled values and performance across different cognitive domains. However, separating the contributions of the visual search and working memory components would provide a more granular understanding of how each PSI measure specifically relates to the observed connectivity findings. This limitation restricts the ability to assess the differential impact of these subtests on connectivity patterns. Future studies that include detailed subtest scores or raw data would offer more nuanced insights into the specific cognitive functions contributing to TBI‐related changes in brain connectivity. Lastly, no significant clusters were identified between orthopedic controls and mTBI participants across the 6‐month period. Several factors may contribute to these null results. One potential explanation lies in the issue of test–retest reliability, which we hypothesize could be a significant factor. Previous research has demonstrated that functional connectivity measures in fMRI often exhibit relatively poor test–retest reliability, even in large cohort studies (Noble et al. 2019). Additionally, the heterogeneous recovery trajectories observed in individuals with mTBI may further complicate group‐level analyses of longitudinal data. Given the substantial variability in recovery outcomes, where some individuals experience significant recovery and others do not, it is plausible that group‐level analyses may struggle to detect common patterns of change.

### 4.4 Conclusion

Advanced functional neuroimaging techniques and data‐driven analyses can increase our understanding of network reorganization following mTBI. Alterations in network connectivity patterns display widespread changes, underscoring mTBI as a disorder that affects the brain globally rather than isolated areas (Palacios et al. 2017; Stevens et al. 2012). In addition, our results indicate that performing imaging can reveal critical information about the complexity and interindividual heterogeneity of injury that appears important in understanding cognitive recovery trajectories. Future longitudinal and interventional studies could leverage these results to create better prognostic models and optimize intervention development.

## Author Contributions

**G.E.‐I.:** conception and design of the study, analysis of data, and drafting a significant portion of the manuscript or figures. **E.M.T.:** analysis of data. **M.A.:** analysis of data. **M.N.:** analysis of data. **T.P.M.:** conception and design of the study, analysis of data, and drafting a significant portion of the manuscript or figures.

## Conflicts of Interest

The authors declare no conflicts of interest.

## Supporting information


Data S1.

## Data Availability

The data that support the findings of this study are available from NIH‐supported Federal Interagency Traumatic Brain Injury Res. Restrictions apply to the availability of these data, which were used under license for this study. Data are available from the author(s) with the permission of NIH‐supported Federal Interagency Traumatic Brain Injury Res.
