# Supplementary material for: Functional Connectivity Patterns Following Mild Traumatic Brain Injury and the Association With Longitudinal Cognitive Function
Source: Hum Brain Mapp. 2025 May 27;46(8):e70237. doi: 10.1002/hbm.70237 (PMC12107601; doi:10.1002/hbm.70237)
Supplement: Supplementary file 1 — Data S1. [file HBM-46-e70237-s001.docx]

**Supplementary material**

Functional connectivity patterns following a traumatic brain injury.

1. Comparison of participant demographics between scanner types.

|  | **Siemens**  **(mean ± SD)** | **GE**  **(mean ± SD)** | **Phillips**  **(mean ± SD)** | **Difference** | ***p*value** |
| --- | --- | --- | --- | --- | --- |
| **Age** | 40.50 ± 17.2 | 34.80 ± 13.70 | 38.80 ± 15.60 | F =5.667 | **0.003*** |
| **Biological Sex** | 147M, 69F | 96M, 51F | 78M, 42F | χ2=0.504 | 0.777 |
| **Education** | 13.20 ± 2.35 | 12.60 ± 2.68 | 14.00 ± 2.07 | F =10.54 | **<0.001*** |
| **Household Income** | $25,000 to $34,999 | $25,000 to $34,999 | $25,000 to $34,999 | F =0.979 | 0.377 |

**Supplementary Table S1**. The ANOVA for age and education revealed a significant effect of scanner type. However, the ANOVA for Household Income did not show a significant effect. Additionally, the Chi-squared test for biological sex distribution across scanner types was not significant. SD, standard deviation.

1. Table with significant cluster. values extracted from the Multivariate Pattern Analysis (MVPA) between cases (mTBI) and controls (orthopedic).

| Cluster | Label | x | y | z | size | size p-FWER | size p-FDR | size p-unc | peak p-FWE | peak p-unc |
| --- | --- | --- | --- | --- | --- | --- | --- | --- | --- | --- |
| Cluster 1 | Occipital Cortex | 30 | -86 | 26 | 698 | 0.000000 | 0.000000 | 0.000000 | 0.528557 | 0.000006 |
| Cluster 2 | Paracingulate Gyrus | 2 | 28 | 40 | 253 | 0.000005 | 0.000002 | 0.000000 | 0.978265 | 0.000042 |
| Cluster 3 | Inferior Temporal gyrus | -54 | -46 | -12 | 119 | 0.003054 | 0.000732 | 0.000063 | 0.682991 | 0.000010 |
| Cluster 4 | Thalamus | 22 | -22 | -6 | 117 | 0.003404 | 0.000732 | 0.000070 | 0.898963 | 0.000023 |
| Cluster 5 | Anterior Cingulate Cortex | 20 | 22 | 26 | 66 | 0.068586 | 0.012204 | 0.001453 | 0.677198 | 0.000010 |
| Cluster 6 | Frontal pole (right) | 8 | 64 | -20 | 54 | 0.148734 | 0.023050 | 0.003293 | 0.998960 | 0.000084 |
| Cluster 7 | Cerebellum | -50 | -58 | -32 | 46 | 0.249834 | 0.035269 | 0.005878 | 0.999951 | 0.000131 |
| Cluster 8 | Frontal pole/Superior Frontal (left) | -8 | 50 | 50 | 40 | 0.364906 | 0.048737 | 0.009283 | 0.867739 | 0.000020 |

**Supplementary Table S2**. MVPA cases vs controls results. FWER, family-wise error rate; FDR, false discovery rate.

1. Replication of Multivariate Pattern Analysis with General Electric (GE) sample (n=155, mTBI=125, Controls=30).


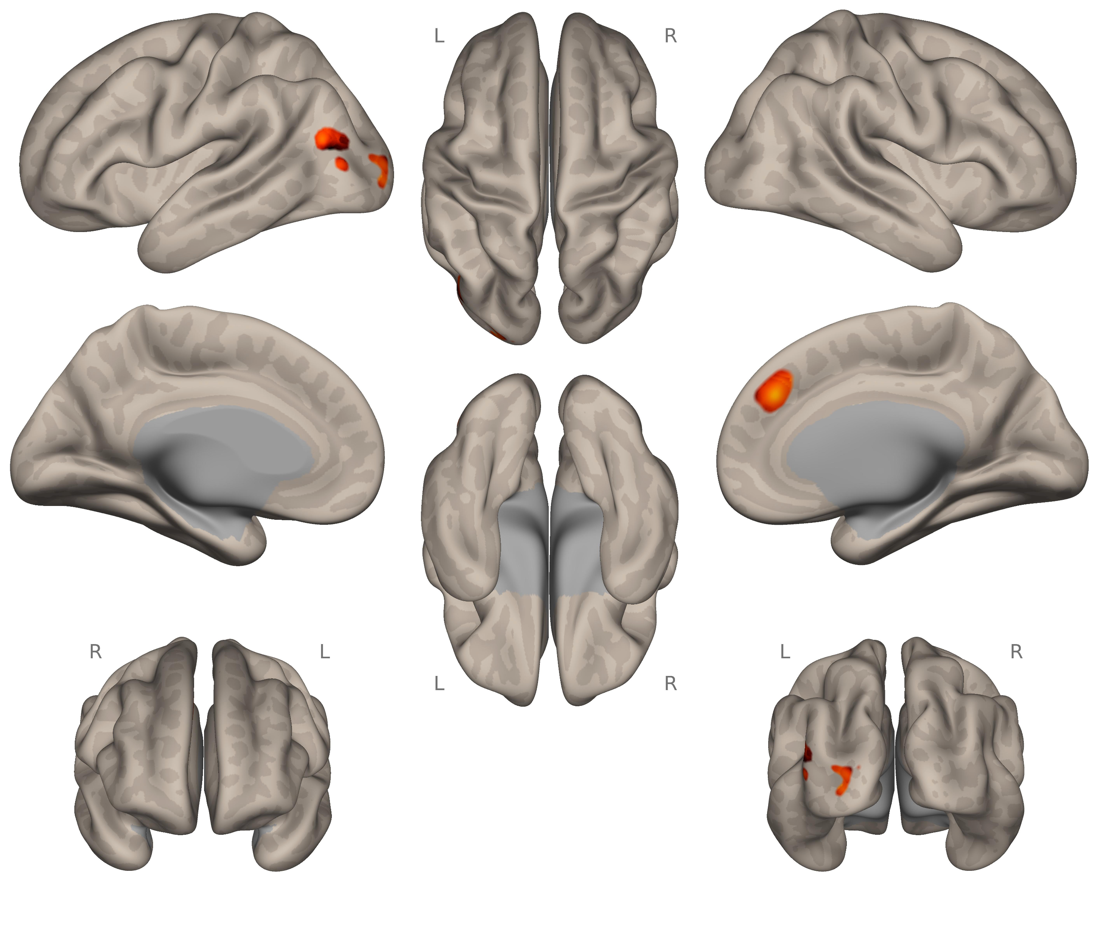


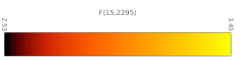


**Supplementary Figure S1**. Representation of MVPA in GE sub-sample replicate.

| Cluster | Label | x | y | z | size | size p-FWER | size p-FDR | size p-unc | peak p-FWE | peak p-unc |
| --- | --- | --- | --- | --- | --- | --- | --- | --- | --- | --- |
| Cluster 1 | Paracingulate Gyrus/Superior Frontal Gyrus | 10 | 40 | 36 | 158 | 0.000050 | 0.000047 | 0.000001 | 0.459552 | 0.000004 |
| Cluster 2 | Lateral Occipital Cortex | -42 | -72 | 22 | 74 | 0.013230 | 0.006164 | 0.000209 | 0.994654 | 0.000044 |
| Cluster 3 | Hippocampus | 32 | -24 | -10 | 59 | 0.042739 | 0.013476 | 0.000685 | 0.094644 | 0.000000 |
| Cluster 4 | Occipital Pole | -28 | -98 | 8 | 42 | 0.174141 | 0.044273 | 0.003002 | 0.960020 | 0.000025 |
| Cluster 5 | Supplementary Motor Area | -4 | -6 | -20 | 39 | 0.223653 | 0.046864 | 0.003972 | 0.979756 | 0.000031 |

**Supplementary Table 3**. MVPA replicate in GE sub-sample. FEW, family-wise error rate; FDR, false discovery rate.

1. Replication of Multivariate Pattern Analysis with Phillips sample (n=166, mTBI=149, Controls=17).


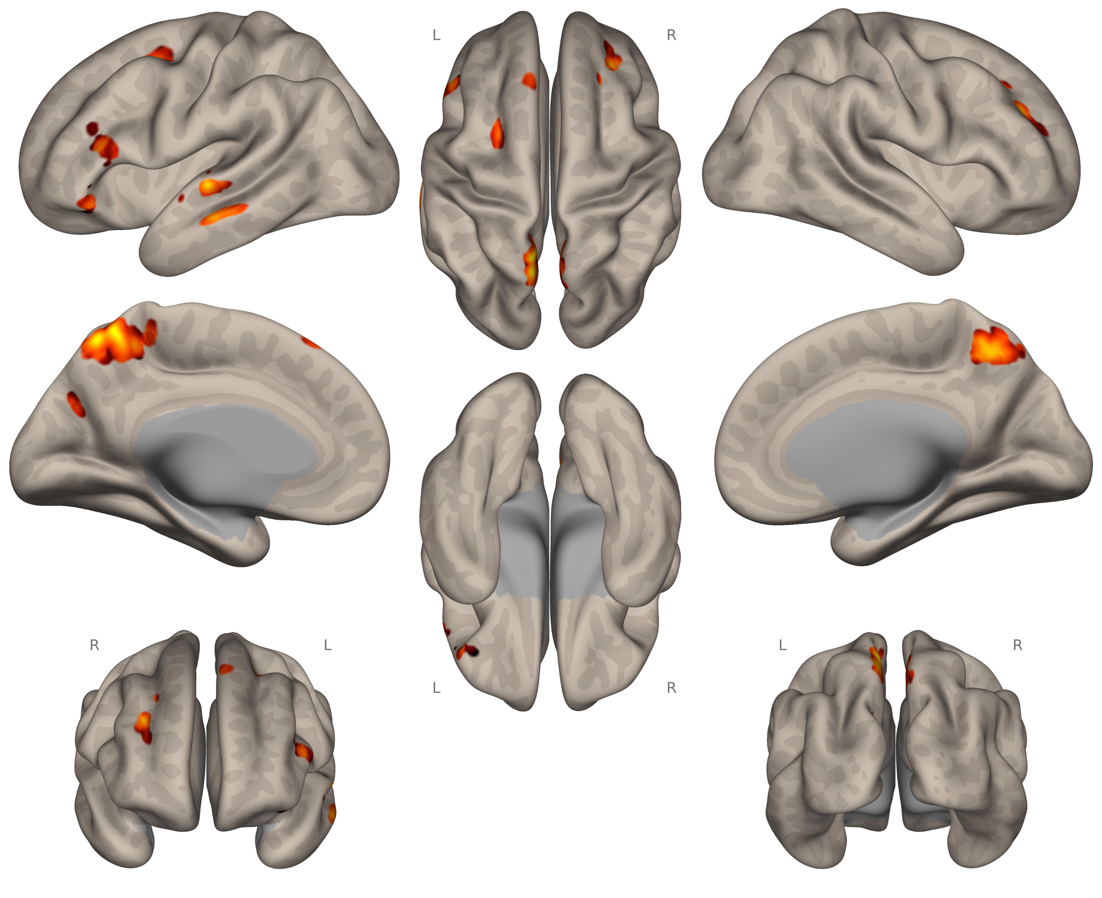


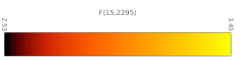


**Supplementary Figure S2**. Representation of MVPA in Phillips sub-sample replicate.

| Cluster | Label | x | y | z | size | size p-FWER | size p-FDR | size p-unc | peak p-FWE | peak p-unc |
| --- | --- | --- | --- | --- | --- | --- | --- | --- | --- | --- |
| Cluster 1 | Precuneus | -4 | -62 | 56 | 782 | 0.000000 | 0.000000 | 0.000000 | 0.244591 | 0.000002 |
| Cluster 2 | Middle Temporal Gyrus | -66 | -16 | -6 | 269 | 0.000001 | 0.000000 | 0.000000 | 0.062618 | 0.000000 |
| Cluster 3 | Superior Frontal Gyrus | -16 | 8 | 46 | 184 | 0.000066 | 0.000013 | 0.000001 | 0.360046 | 0.000003 |
| Cluster 4 | Frontal Pole | 26 | 32 | 26 | 174 | 0.000108 | 0.000016 | 0.000002 | 0.358556 | 0.000003 |
| Cluster 5 | Pars Triangularis | -48 | 28 | 14 | 87 | 0.013650 | 0.001624 | 0.000262 | 0.578771 | 0.000007 |
| Cluster 6 | Frontal Orbital Cortex | -48 | 30 | -12 | 52 | 0.139976 | 0.014851 | 0.002874 | 0.979706 | 0.000039 |
| Cluster 7 | Precuneus | -18 | -70 | 28 | 36 | 0.416194 | 0.043287 | 0.010259 | 0.999962 | 0.000124 |
| Cluster 8 | Superior Frontal Gyrus | -8 | 34 | 56 | 35 | 0.443480 | 0.043287 | 0.011171 | 0.999920 | 0.000113 |

**Supplementary Table 4**. MVPA replicate in Phillips sub-sample. FEW, family-wise error rate; FDR, false discovery rate.

1. Linear mixed effects models with cognition, rs-fMRI, time and time interaction.

| **Association between rs-fMRI and TMT (Trail Making Test) A over time** | | | | |
| --- | --- | --- | --- | --- |
|  | *B* | *p* | CI | Conditional R2 |
| Occipital Cortex +30 -86 +26:timepoint | -3.339 | 0.621 | -16.6512 – 9.972 | 0.654 |
| Paracingulate +02 +28 +40:timepoint | 0.774 | 0.855 | -7.535 – 9.083 | 0.646 |
| Inferior Temporal -54 -46 -12:timepoint | -12.267 | **0.002*** | -20.243 – -4.291 | 0.675 |
| ACC +20 +22 +26 :timepoint | 2.826 | 0.588 | -7.437 – 13.089 | 0.658 |
| Frontal Pole +08 +64 -20:timepoint | -4.125 | 0.256 | -11.252 – 3.002 | 0.657 |
| Superior Frontal -08 +50 +50:timepoint | -5.372 | 0.212 | -13.819 – 3.076 | 0.654 |
| Thalamus +22 -22 +06:timepoint | -6.621 | 0.189 | -16.515 – 3.272 | 0.674 |
| Cerebellum -50 -58 -32:timepoint | 13.985 | **0.000*** | 5.676 - 22.295 | 0.677 |
| **Association between rs-fMRI and TMT (Trail Making Test) B over time** | | | | |
|  | *B* | *p* | CI | Conditional R2 |
| Occipital Cortex +30 -86 +26:timepoint | -10.228 | 0.543 | -43.283 – 22.828 | 0.762 |
| Paracingulate +02 +28 +40:timepoint | -16.873 | 0.111 | -37.624 – 3.878 | 0.762 |
| Inferior Temporal -54 -46 -12:timepoint | 6.836 | 0.511 | -13.590 – 27.263 | 0.760 |
| ACC +20 +22 +26 :timepoint | -35.953 | 0.005 | -61.272 – -10.635 | 0.774 |
| Frontal Pole +08 +64 -20:timepoint | -25.904 | **0.004*** | -43.526 – -8.282 | 0.774 |
| Superior Frontal -08 +50 +50:timepoint | -6.137 | 0.548 | -26.205 – 13.931 | 0.764 |
| Thalamus +22 -22 +06:timepoint | -5.424 | 0.670 | -30.458 – 19.611 | 0.760 |
| Cerebellum -50 -58 -32:timepoint | 10.796 | 0.314 | -10.270 – 31.861 | 0.759 |

| **Association between rs-fMRI and TMT B-A over time** | | | | | | | | |  |
| --- | --- | --- | --- | --- | --- | --- | --- | --- | --- |
|  | *B* | | | *p* | CI | | | Conditional R2 | |
| Occipital Cortex +30 -86 +26:timepoint | -6.603 | | | 0.680 | -38.086 – 24.879 | | | 0.669 | |
| Paracingulate +02 +28 +40:timepoint | -17.095 | | | 0.089 | -36.798 – 2.609 | | | 0.670 | |
| Inferior Temporal -54 -46 -12:timepoint | 19.945 | | | 0.044 | 0.571 – 39.319 | | | 0.665 | |
| ACC +20 +22 +26 :timepoint | -38.625 | | | 0.002 | -62.653 – -14.597 | | | 0.681 | |
| Frontal Pole +08 +64 -20:timepoint | -21.213 | | | **0.014*** | -38.069 – -4.358 | | | 0.680 | |
| Superior Frontal -08 +50 +50:timepoint | -1.989 | | | 0.838 | -21.086 – 17.107 | | | 0.667 | |
| Thalamus +22 -22 +06:timepoint | 0.410 | | | 0.973 | -23.426 – 24.246 | | | 0.657 | |
| Cerebellum -50 -58 -32:timepoint | -3.948 | | | 0.701 | -24.155 – 16.259 | | | 0.665 | |
| **Association between rs-fMRI and WAIS (Wechsler Adult Intelligence Scale) over time** | | | | | | | | |  |
|  | | *B* | *p* | | | CI | Conditional R2 | |  |
| Occipital Cortex +30 -86 +26:timepoint | | 0.925 | 0.423 | | | -1.343 – 3.192 | 0.822 | |  |
| Paracingulate +02 +28 +40:timepoint | | -0383 | 0.592 | | | -1.784 – 1.019 | 0.831 | |  |
| Inferior Temporal -54 -46 -12:timepoint | | 0.432 | 0.538 | | | -0.948 – 1.813 | 0.831 | |  |
| ACC +20 +22 +26 :timepoint | | 0.260 | 0.771 | | | -1.497 – 2.017 | 0.822 | |  |
| Frontal Pole +08 +64 -20:timepoint | | 0.019 | 0.975 | | | -1.199 – 1.237 | 0.822 | |  |
| Superior Frontal -08 +50 +50:timepoint | | 0.724 | 0.300 | | | -0.650 – 2.099 | 0.823 | |  |
| Thalamus +22 -22 +06:timepoint | | 0.694 | 0.420 | | | -0.991 – 2.386 | 0.823 | |  |
| Cerebellum -50 -58 -32:timepoint | | -1.052 | 0.149 | | | -2.484 – 0.379 | 0.829 | |  |

| **Association between rs-fMRI and RAVLT (Rey Auditory Verbal Learning Test) total sum over time** | | | | |
| --- | --- | --- | --- | --- |
|  | *B* | *p* | CI | Conditional R2 |
| Occipital Cortex +30 -86 +26:timepoint | -1.762 | 0.739 | -12.172 – 8.647 | 0.676 |
| Paracingulate +02 +28 +40:timepoint | 0.167 | 0.959 | -6.213 – 6.548 | 0.682 |
| Inferior Temporal -54 -46 -12:timepoint | -4.504 | 0.161 | -10.811 – 1.803 | 0.678 |
| ACC +20 +22 +26 :timepoint | 2.913 | 0.474 | -5.093 – 10.919 | 0.666 |
| Frontal Pole +08 +64 -20:timepoint | 0.786 | 0.780 | -4.758 – 6.330 | 0.674 |
| Superior Frontal -08 +50 +50:timepoint | -1.271 | 0.690 | -7.546 – 5.004 | 0.679 |
| Thalamus +22 -22 +06:timepoint | 5.380 | 0.171 | -2.341 – 13.102 | 0.679 |
| Cerebellum -50 -58 -32:timepoint | -3.615 | 0.280 | -10.191 – 2.960 | 0.668 |
| **Association between rs-fMRI and RAVLT (Rey Auditory Verbal Learning Test) learnt over time** | | | | |
|  | *B* | *p* | CI | Conditional R2 |
| Occipital Cortex +30 -86 +26:timepoint | 3.825 | 0.019 | -0.280 – 6.394 | 0.259 |
| Paracingulate +02 +28 +40:timepoint | -0.482 | 0.632 | -2.465 – 1.500 | 0.248 |
| Inferior Temporal -54 -46 -12:timepoint | -0.162 | 0.871 | -2.135 – 1.809 | 0.249 |
| ACC +20 +22 +26 :timepoint | -1.005 | 0.426 | -3.489 – 1.478 | 0.251 |
| Frontal Pole +08 +64 -20:timepoint | -0.012 | 0.988 | -1.733 – 1.708 | 0.253 |
| Superior Frontal -08 +50 +50:timepoint | -1.953 | 0.047 | -3.880 – -0.025 | 0.261 |
| Thalamus +22 -22 +06:timepoint | 2.015 | 0.097 | -0.365 – 4.396 | 0.257 |
| Cerebellum -50 -58 -32:timepoint | -0.233 | 0.824 | -2.289 – 1.823 | 0.248 |

**Supplementary Table S5**. Full linear mixed-effects model for each cognitive test. Beta coefficients represent the interaction term between *time*functional connectivity.* *Indicates a significant effect at p < 0.05 for both the main correlation between the cognitive test scores and functional connectivity of the cluster, as well as for the interaction effect within the same model.


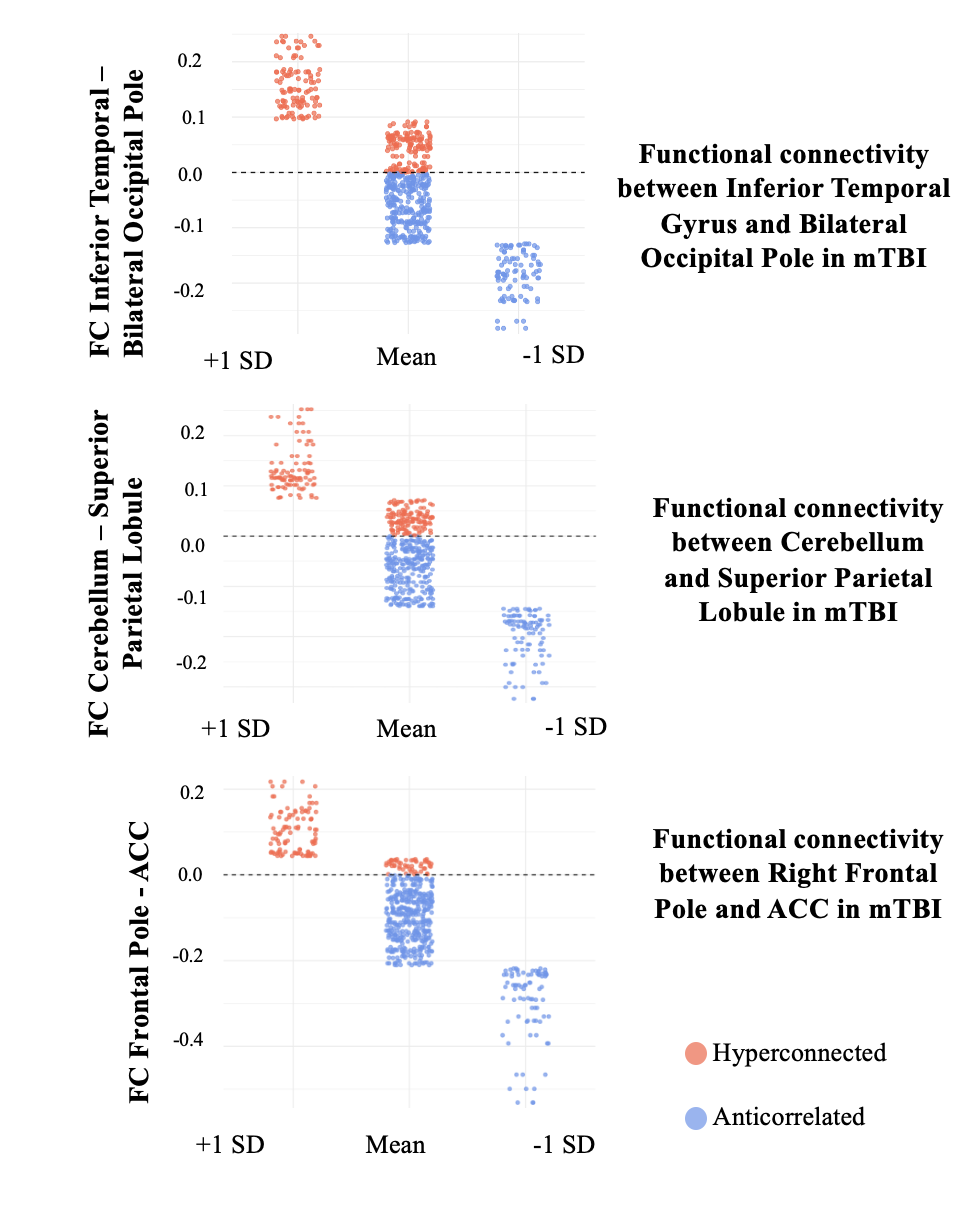


**Supplementary Figure S3.** Boxplot demonstrating the interindividual variability in the functional connectivity between these nodes in the TBI group, where some individuals with TBI show hyperconnectivity and others hypoconnectivity (the group-level result). Individuals are grouped by taking the mean, +1SD, and –1 SD as part of the marginal effect analysis. FC, functional connectivity; ACC, anterior cingulate cortex; SD, standard deviation.

1. Exploratory analysis of sex differences between cases and controls in the Siemens dataset.

| Cluster | Label | x | y | z | size | size p-FWER | size p-FDR | size p-unc | peak p-FWE | peak p-unc |
| --- | --- | --- | --- | --- | --- | --- | --- | --- | --- | --- |
| Cluster 1 | Lateral Occipital Cortex | +28 | -84 | +12 | 419 | 0.000000 | 0.000000 | 0.000000 | 0.927937 | 0.000028 |
| Cluster 2 | Paracingulate Gyrus | 02 | 22 | 46 | 158 | 0.000520 | 0.000171 | 0.000011 | 0.997501 | 0.000074 |
| Cluster 3 | Thalamus | 18 | -22 | -08 | 115 | 0.00486 | 0.001005 | 0.000097 | 0.734218 | 0.000012 |
| Cluster 4 | Inferior/Middle Temporal Gyrus | -54 | -46 | -14 | 71 | 0.056608 | 0.009559 | 0.001233 | 0.959435 | 0.000035 |
|  |  |  |  |  |  |  |  |  |  |  |

**Supplementary Table S6**. MVPA results comparing cases versus controls and examining sex differences FWER, family-wise error rate; FDR, false discovery rate.


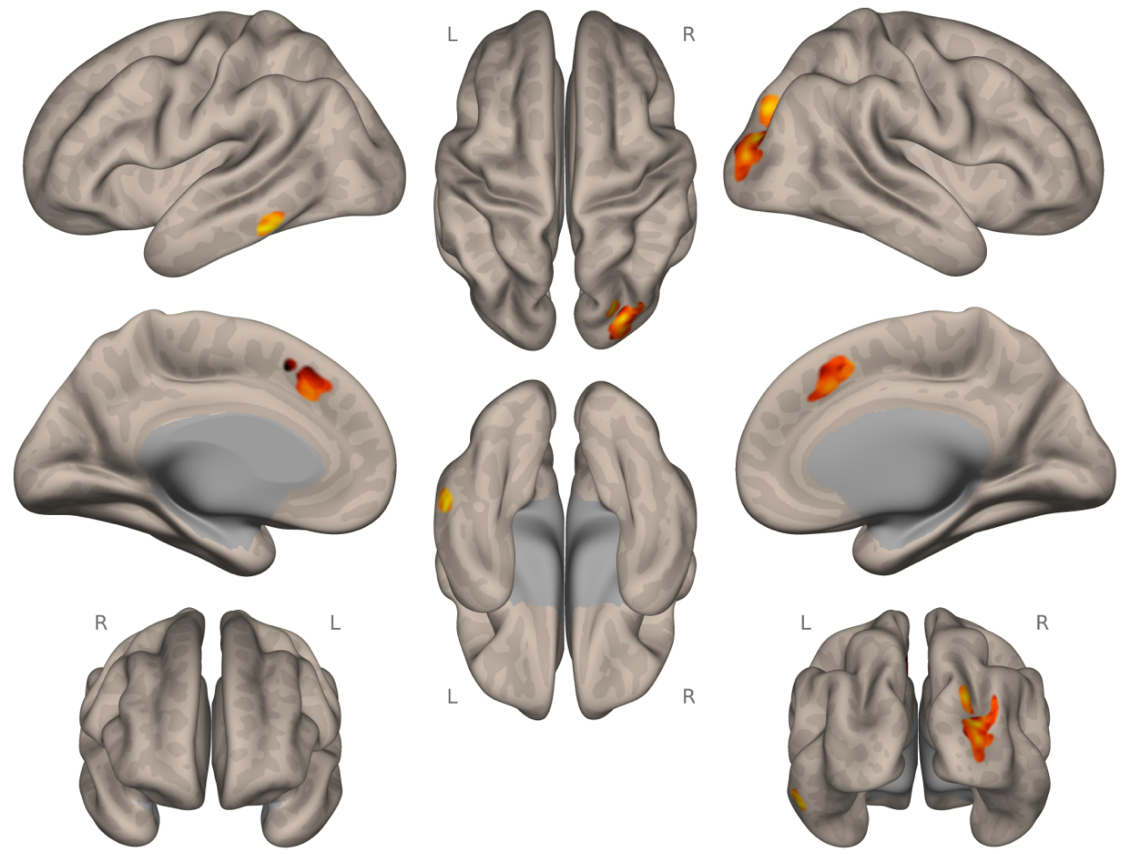


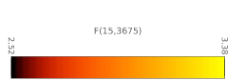


**Supplementary Figure S4** Visualization of MVPA sex differences in the Siemens sub-sample.


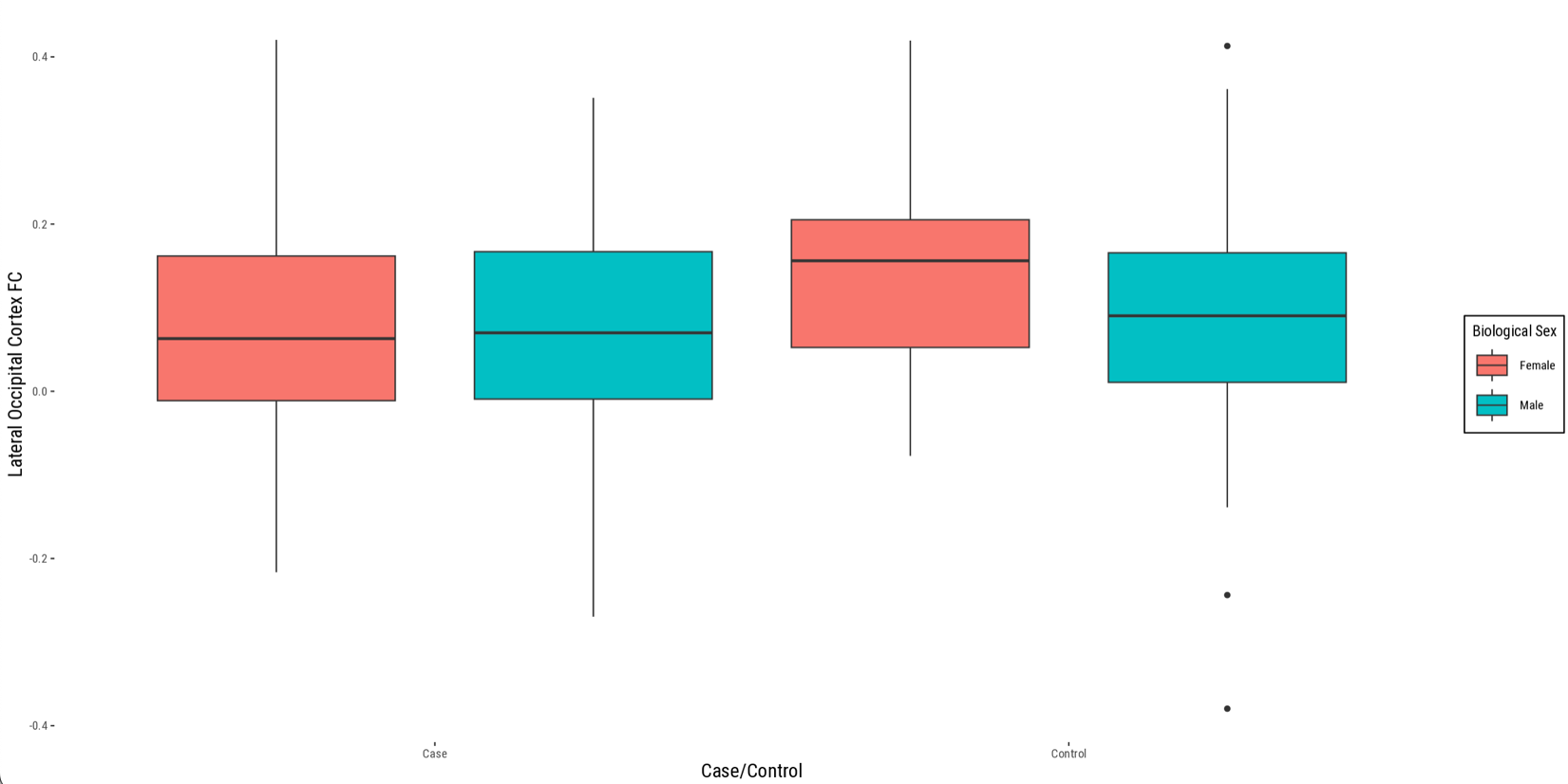


**Supplementary Figure S5** Bar plots depicting functional connectivity differences in the lateral occipital cortex between cases and controls, as well as between males and females.


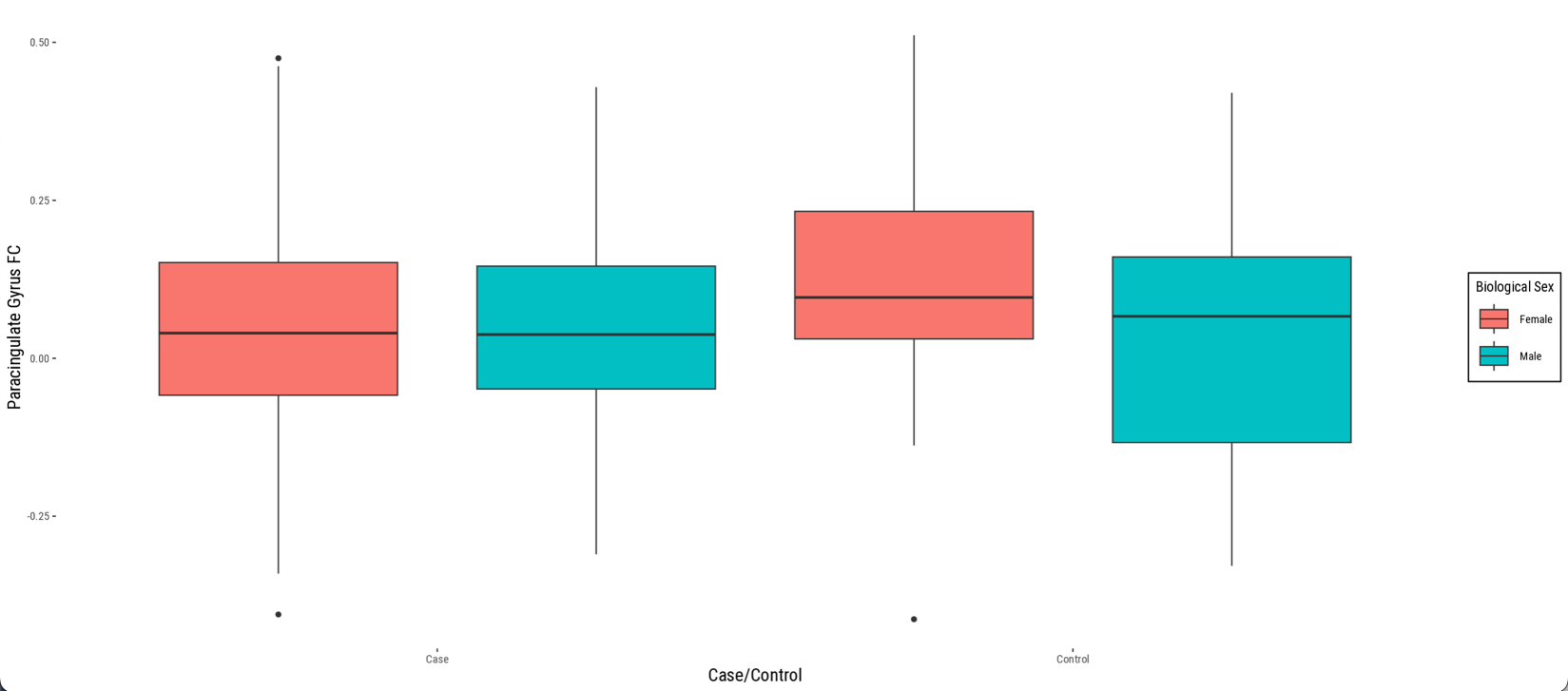


**Supplementary Figure S6** Bar plots depicting functional connectivity differences in the paracingulate gyrus between cases and controls, as well as between males and females.


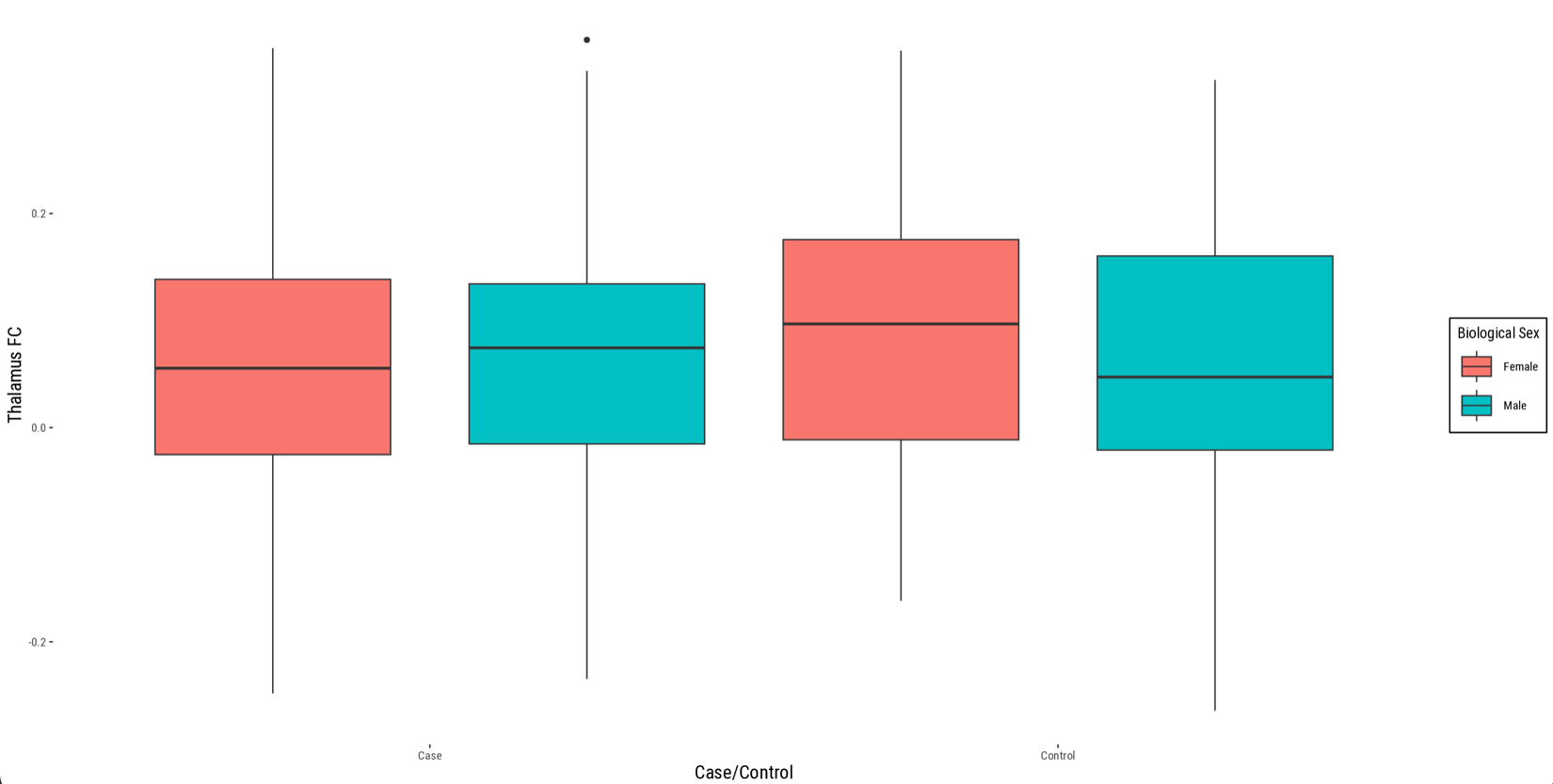


**Supplementary Figure S7** Bar plots depicting functional connectivity differences in the thalamus between cases and controls, as well as between males and females.


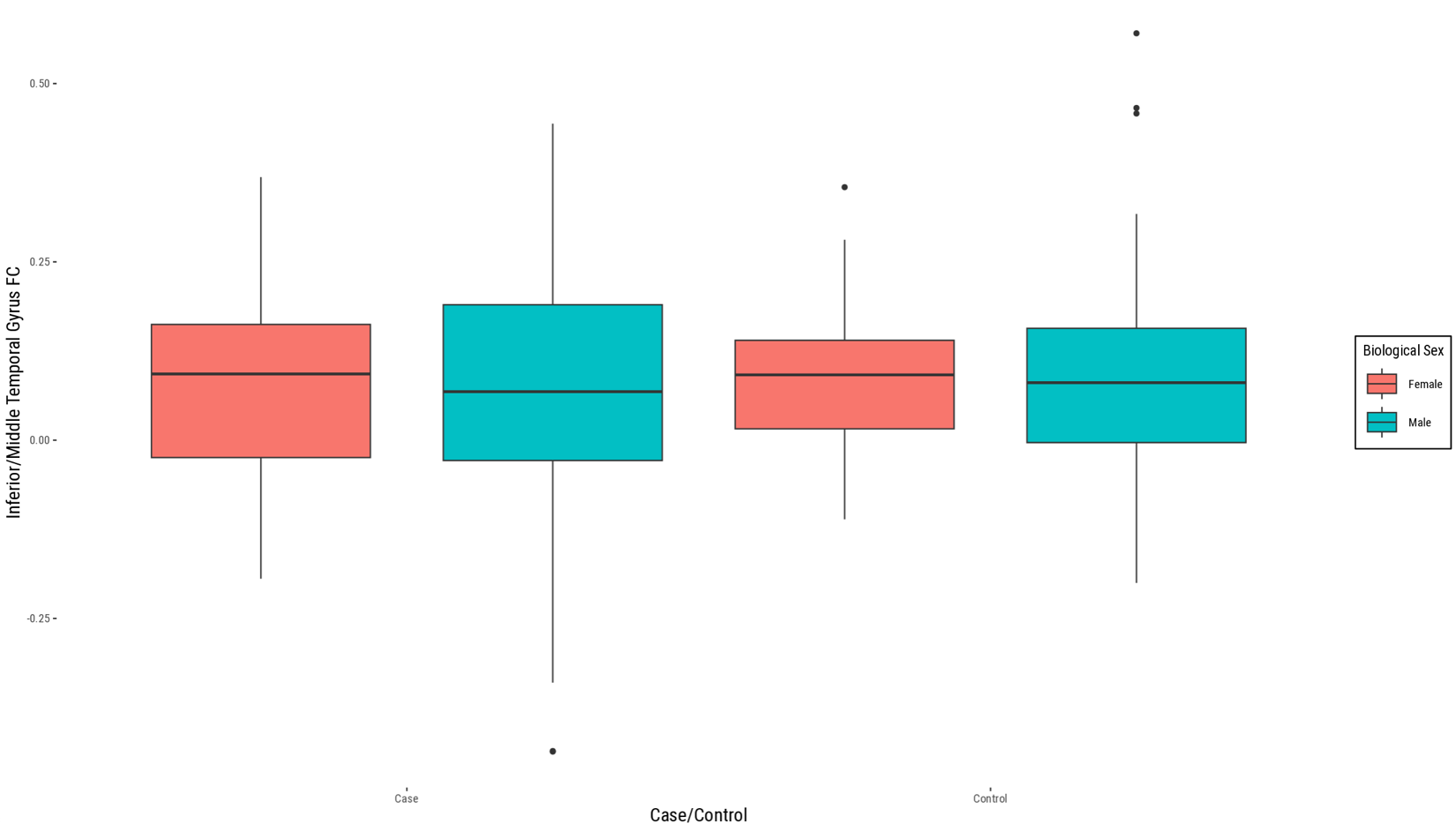


**Supplementary Figure S8** Bar plots depicting functional connectivity differences in the inferior/middle temporal gyrus between cases and controls, as well as between males and females.
